# Comprehensive analyses of competing endogenous RNA networks reveal potential biomarkers for predicting hepatocellular carcinoma recurrence

**DOI:** 10.1186/s12885-021-08173-0

**Published:** 2021-04-20

**Authors:** Ping Yan, Zuotian Huang, Tong Mou, Yunhai Luo, Yanyao Liu, Baoyong Zhou, Zhenrui Cao, Zhongjun Wu

**Affiliations:** Department of Hepatobiliary Surgery, The First Affiliated Hospital of Chongqing Medical University, Chongqing, 400016 People’s Republic of China

**Keywords:** Hepatocellular carcinoma, Recurrence, Competing endogenous RNA, Prognosis, Biomarker

## Abstract

**Background:**

Hepatocellular carcinoma (HCC) is one of the most common and deadly malignant tumors, with a high rate of recurrence worldwide. This study aimed to investigate the mechanism underlying the progression of HCC and to identify recurrence-related biomarkers.

**Methods:**

We first analyzed 132 HCC patients with paired tumor and adjacent normal tissue samples from the Gene Expression Omnibus (GEO) database to identify differentially expressed genes (DEGs). The expression profiles and clinical information of 372 HCC patients from The Cancer Genome Atlas (TCGA) database were next analyzed to further validate the DEGs, construct competing endogenous RNA (ceRNA) networks and discover the prognostic genes associated with recurrence. Finally, several recurrence-related genes were evaluated in two external cohorts, consisting of fifty-two and forty-nine HCC patients, respectively.

**Results:**

With the comprehensive strategies of data mining, two potential interactive ceRNA networks were constructed based on the competitive relationships of the ceRNA hypothesis. The ‘upregulated’ ceRNA network consists of 6 upregulated lncRNAs, 3 downregulated miRNAs and 5 upregulated mRNAs, and the ‘downregulated’ network includes 4 downregulated lncRNAs, 12 upregulated miRNAs and 67 downregulated mRNAs. Survival analysis of the genes in the ceRNA networks demonstrated that 20 mRNAs were significantly associated with recurrence-free survival (RFS). Based on the prognostic mRNAs, a four-gene signature (ADH4, DNASE1L3, HGFAC and MELK) was established with the least absolute shrinkage and selection operator (LASSO) algorithm to predict the RFS of HCC patients, the performance of which was evaluated by receiver operating characteristic curves. The signature was also validated in two external cohort and displayed effective discrimination and prediction for the RFS of HCC patients.

**Conclusions:**

In conclusion, the present study elucidated the underlying mechanisms of tumorigenesis and progression, provided two visualized ceRNA networks and successfully identified several potential biomarkers for HCC recurrence prediction and targeted therapies.

**Supplementary Information:**

The online version contains supplementary material available at 10.1186/s12885-021-08173-0.

## Background

Liver cancer was reported to be the sixth most common cancer and the fourth leading cause of cancer-related death in the world according to global cancer statistics in 2018 [1]. In the United States, approximately 42,030 people are diagnosed with liver cancer, and 31,780 die annually according to the latest cancer statistics in 2019 [2]. Hepatocellular carcinoma (HCC) is the main type of primary liver cancer, comprising 75–85% of cases [3]. Despite the fact that the diagnostic approaches and therapeutic efficacy of HCC have gradually improved, the majority of patients with HCC are still diagnosed at an advanced stage with severe hepatic dysfunction due to the asymptomatic nature of the disease. Accordingly, the 5-year overall survival (OS) and recurrence-free survival (RFS) rates of advanced-stage HCC patients remain extremely low, and approximately 70% of HCC patients experience recurrence or extrahepatic metastasis within 5 years [4, 5]. Considering the poor outcomes, many researchers have sought to identify prognostic factors based on clinicopathological and molecular features to help increase life expectancy and improve quality of life. However, more reliable biomarkers associated with the molecular mechanisms that mediate prognosis remain to be deeply explored for early diagnosis and optimized therapy.

It has been reported that less than 2% of the total genome encodes protein-coding genes, so research on noncoding RNA transcripts, including long noncoding RNAs (lncRNAs), microRNAs (miRNAs) and circular RNAs (circRNAs), has become increasingly popular [6]. In recent years, emerging evidence has indicated that lncRNAs, which consist of more than 200 nucleotides, play a vital role in a large variety of biological processes, including genetic transcription, chromosome modification, cell cycle, cell differentiation and migration [7–9]. Numerous studies have shown that miRNAs, which consist of approximately 22 nucleotides, may participate in tumor initiation, progression, and invasion by post-transcriptionally downregulating target gene expression by complementation to miRNA response elements (MREs) on messenger RNA (mRNA) [10–12]. Moreover, the competing endogenous RNA (ceRNA) hypothesis proposed by Salmena et al. [13] depicted a molecular biological regulatory mechanism for posttranscriptional regulation in which ceRNAs can act as miRNA sponges and inhibit miRNA function by competitively binding to MREs on a target mRNA. Thereafter, numerous experiments have validated the hypothesis that this type of indirect regulatory mechanism is involved in tumorigenesis and progression [14–16]. Several studies on ceRNAs have reported valuable factors for predicting the OS of HCC patients [17–19]; however, the molecular biological mechanisms underlying the occurrence, progression, recurrence and metastasis of HCC have not yet been fully illuminated, especially the molecular mechanisms that mediate recurrence, which remain unclear and require further investigation.

In this study, microarray and sequencing data were collected from a large sample size of patients with HCC in the Gene Expression Omnibus (GEO) and The Cancer Genome Atlas (TCGA) databases and applied to identify differentially expressed genes (DEGs) in HCC. Two predictable ceRNA networks, including the ‘upregulated’ network and the ‘downregulated’ network, were then constructed based on the ceRNA hypothesis. Twenty mRNAs involved in the ceRNA networks were identified as recurrence-related genes. Furthermore, LASSO-penalized regressions were utilized to screen the recurrence-related genes and successfully establish a prognostic signature consisting of ADH4, DNASE1L3, HGFAC and MELK. More importantly, a quantitative real-time PCR method was adopted to verify this signature in an external cohort, which showed good predictive performance. These comprehensive analyses aimed to reveal the underlying molecular regulatory mechanisms of HCC tumorigenesis and progression and develop a prognostic signature that can be used to predict the RFS of HCC patients.

## Materials and methods

### Data retrieval and mining

**Fig. 1:**
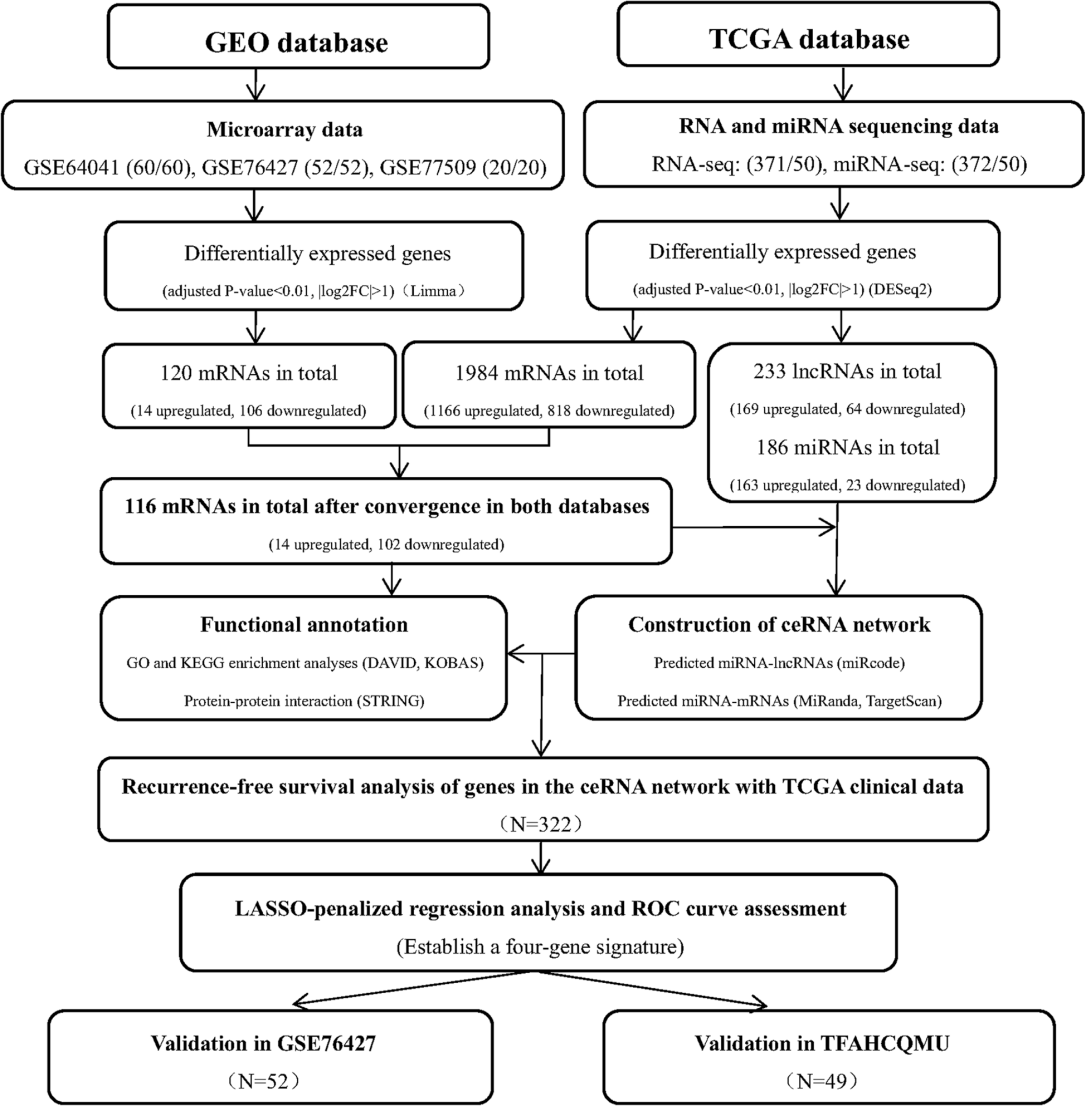
Flowchart of data collection and method implementation in this study

Three microarray datasets, GSE64041 [20], GSE76427 [21] and GSE77509 [22], were obtained from the GEO database (https://www.ncbi.nlm.nih.gov/geo/). Considering that small sample sizes are one of the most challenging factors in microarray analysis, datasets consisting of at least 20 samples were chosen for download. To provide more reliable results, only HCC patients with paired tumor and adjacent normal tissue samples in the dataset were employed to screen the DEGs. Finally, a total of 132 HCC patients, including 60, 52, and 20 pairs of samples from GSE64041, GSE76427, and GSE7509, respectively, were selected. We also downloaded the RNA sequencing (RNA-seq) and microRNA sequencing (miRNA-seq) data of 372 HCC patients, containing 371 RNA-seq and 372 miRNA-seq data points of tumor samples and 50 RNA-seq and 50 miRNA-seq data points of adjacent normal samples, from the TCGA database (https://portal.gdc.cancer.gov/). All datasets in the current study were obtained from public databases, including the GEO and TCGA databases, which allowed researchers to download data for scientific purposes; thus, ethics approval was not required. A flowchart of the data collection process and method implementation is presented in Fig. 1.

Forty-nine paired HCC tumor tissues and adjacent normal tissues were obtained between January 2015 and April 2016 from the First Affiliated Hospital of Chongqing Medical University (the TFAHCQMU cohort). All of the diagnoses were confirmed by two experienced pathologists. This research was approved by the Ethics Committee of the First Affiliated Hospital of Chongqing Medical University. All the clinicopathological features of the HCC patients with complete recurrence survival time from the TCGA cohort and the TFAHCQMU cohort are presented in Supplementary Table S1.

### Analysis of DEGs

The Limma package for R software (version 4.0.2) [23] was adopted for the normalization and log base 2 transformation of microarray data from the GEO database and the identification of preliminary differentially expressed mRNAs (DEmRNAs) based on the following significance cutoff levels: adjusted *P*-value< 0.01 and |log2 fold change (FC)| > 1. Similarly, in the TCGA database, the DESeq2 package (version 1.26.0) for R software was used to analyze the DEmRNAs, differentially expressed lncRNAs (DElncRNAs) and differentially expressed miRNAs (DEmiRNAs) with adjusted *P*-value< 0.01 and |log2FC| > 1 set as the cutoff criteria. Intersections of uniformly upregulated and downregulated mRNAs in the two databases were used for further functional analyses.

### Functional enrichment analysis

The Database for Annotation, Visualization, and Integrated Discovery (DAVID; version 6.8; http://david.ncifcrf.gov/) [24] was applied to perform the Gene Ontology (GO) functional annotations, including molecular function (MF), biological process (BP) and cellular component (CC) terms, of the intersecting DEmRNAs. The KEGG Orthology Based Annotation System (KOBAS; version 3.0; http://kobas.cbi.pku.edu.cn/) was used to evaluate the Kyoto Encyclopedia of Genes and Genomes (KEGG) pathway enrichment of the DEmRNAs. The Search Tool for the Retrieval of Interacting Genes (STRING; version 11.0; https://string-db.org/) [25] database was adopted to construct the protein-protein interaction (PPI) network, which was then visualized by Cytoscape (version 3.7.1) [26] software. GO functional annotations, KEGG pathways and PPI network, the analyses of which were based on the DEmRNAs, were considered significantly enriched when *P*-value< 0.01, false discovery rate (FDR) < 0.01and the combined score of ≥0.4, respectively.

### Construction of the ceRNA network

The intersections of uniformly upregulated and downregulated DEmRNAs were taken as described previously. DElncRNAs and DEmiRNAs were selected to construct the ceRNA networks. The lncRNA-miRNA interactions were predicted using miRcode (version 11.0; http://www.mircode.org/). The miRNA-mRNA interactions were predicted by the cooperative utilization of TargetScan (version 7.2; http://www.targetscan.org) and miRanda (version 6.0; http://www.miranda.org/). Cytoscape software was finally applied to establish and visualize the interactive ceRNA networks based on the predicted lncRNA-miRNA and miRNA-mRNA interactions. To better understand the potential functions and values of the DEmRNAs in the regulatory ceRNA networks, GO functional annotation, KEGG pathway enrichment and PPI network analyses were performed.

### Survival analysis

Kaplan-Meier survival analysis was utilized to assess the RFS related to the genes in the ceRNA networks with the gene expression profiles of tumor tissues and clinical information of HCC patients from the TCGA database. A log-rank test, dividing all samples into high and low expression groups based on the median expression level of each gene, was performed using R software (version 4.0.2) to evaluate the differences in RFS. According to the log-rank test results, *P*-value< 0.05 was considered to indicate a statistically significant difference. Genes were then regarded as recurrence-related. To better understand the relationship between the recurrence-related genes, “ggExtra” R softwre package was applied to analyze the expression correlation. Pearson correlation analysis was performed with *P*-value< 0.05 being set as significant difference.

### Generation of a prognostic signature

Based on the identified recurrence-related genes, LASSO-penalized Cox regression were performed using R software (version 4.0.2) to screen the candidate mRNAs to establish a multigene prognostic signature with TCGA clinical information. The risk score (RS) was calculated using the sum of the identified recurrence-related gene expression values weighted by the coefficients derived from the LASSO-penalized Cox regression model. The prognostic RS for each patient could be calculated by the following formula: RS = (β1 × expression of gene1) + (β2 × expression of gene2) + ... + (βn × expression of genen). All the samples were categorized into a high-risk group and a low-risk group according to the median value of the RS.

### Performance of the prognostic signature

To evaluate the discrimination and prediction abilities of the RS system, the Kaplan-Meier survival analysis results were assessed in R software, time-dependent receiver operating characteristic (ROC) curve analysis was conducted, and the area under the ROC curve (AUC) was calculated. Moreover, a nomogram was build to investigate the probability of 1-, 2-, 3-, and 5-RFS of HCC. The potential relationships between the prognostic four-gene signature and other clinicopathological features were further investigated. *P*-value< 0.05 was considered to indicate statistical significance.

### External validation of the prognostic signature

Expression at the level of mRNA between paired HCC tumor tissues and adjacent normal tissues of the four genes in the signature was validated in two external cohorts, one consisting of 49 HCC patients from TFAHCQMU and the other 52 HCC patients from GSE76427 dataset in the GEO database [21]. Expression at the level of protein between HCC tumor tissues and normal tissues of the four genes in the signature was validated in the Human Protein Atlas (http://www.proteinatlas.org) online database. The risk score for each included patient was calculated with the same prognostic gene-signature based model. Likewise, the Kaplan-Meier curve and the ROC curve were used to evaluate the predictive performance of the prognostic gene signature. The potential relationships between the prognostic four-gene signature and other clinicopathological features were also analyzed.

### Independence assessment

Univariate and multivariate Cox regression analyses were successively performed with forwarding stepwise procedure to investigate whether the prognostic gene signature or other clinicopathological features, including age, gender, tumor grade, vascular invasion, cirrhosis, and AJCC stage, could be independent factor. *P*-value< 0.05 were considered as statistically significant.

### Establishment of a predictive nomogram

The widely-used nomogram, including all independent factors identified by aforementioned multivariate Cox regression analysis, was constructed and used to predict 1-, 2-, 3-, and 5-year RFS of HCC patients. Performance of the nomogram was assessed by discrimination and calibration. The concordance index was calculated to assess the discrimination of the nomogram and the calibration curve of the nomogram was plotted to observe the nomogram prediction probabilities.

### Real-time PCR

Forty-nine pairs of resected HCC tumor tissues and adjacent normal tissues were preserved at − 80 °C until mRNA extraction by TRIzol (Invitrogen, Carlsbad, CA). Then, GoScript (Promega, Madison, WI) was utilized to perform reverse transcription. Finally, quantitative real-time polymerase chain reaction (qRT-PCR) with TB Green Premix Ex Taq II (Takara, Tokyo, Japan) was applied to analyze the gene expression level, which was normalized to the GAPDH expression level and quantified using the 2^-ΔΔCT^ method. The primers are listed in Supplementary Table S2.

### Statistical analysis

The gene expression differences between the tumor tissues and adjacent normal tissues of patients in the TFAHCQMU cohort were compared using paired Student’s t-tests. Differences in categorical variables, such as the relationships between the prognostic signature and other clinicopathological features, were assessed using the Chi square test. Statistical analysis was conducted using IBM SPSS Statistics (version 25.0) and R software (version 4.0.2).

## Results

### Identification of DEGs

**Fig. 2:**
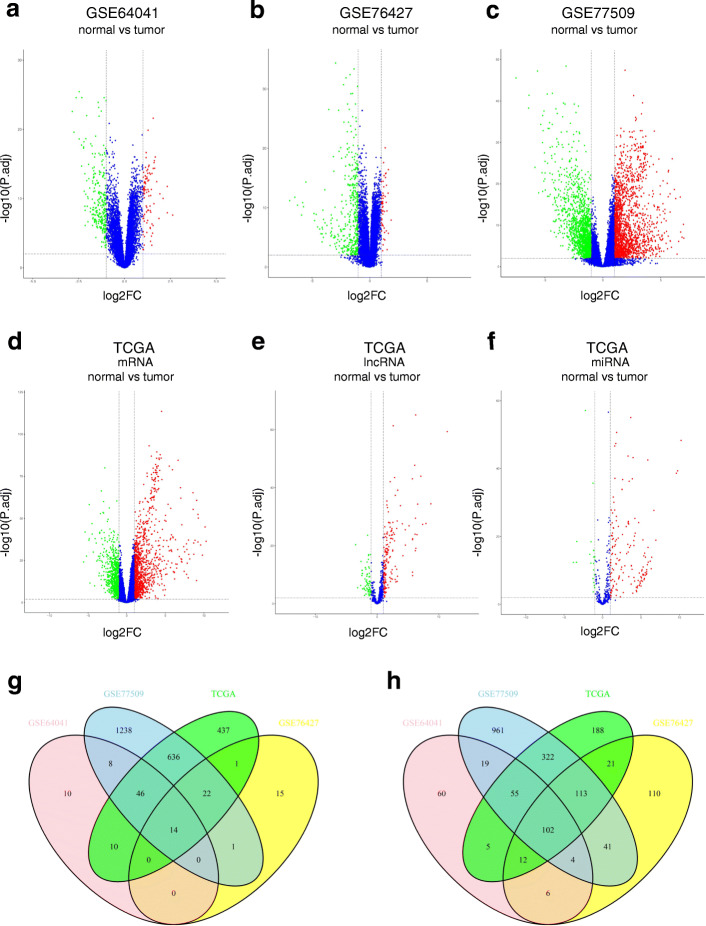
Volcano plots of DEGs in both databases. Volcano plots of DEGs in the GEO database: **a** GSE64041; **b** GSE76427; **c** GSE77509; Volcano plots of DEGs in the TCGA database: **d** DEmRNAs; **e** DElncRNAs; **f** DEmiRNAs; **g** The intersection of upregulated DEmRNAs between the TCGA and GEO databases; **h** The intersection of downregulated DEmRNAs between the TCGA and GEO databases

After integrative analysis of three GEO datasets, a total of 120 DEmRNAs were obtained, including 14 upregulated and 106 downregulated mRNAs. The number of DEmRNAs identified from each dataset is shown in volcano plots (Fig. 2a-c). In the TCGA database, we obtained 1984 DEmRNAs (1166 upregulated and 818 downregulated), 233 DElncRNAs (169 upregulated and 64 downregulated) and 186 DEmiRNAs (163 upregulated and 23 downregulated) (Fig. 2d-f). Heatmaps of the top 200 DEmRNAs based on adjusted *P*-values were created. Overall, 233 DElncRNAs and 186 DEmiRNAs are shown in Figs. S1, S2 and S3. After the convergence of uniformly upregulated and downregulated mRNAs in both the GEO and TCGA databases, 116 DEmRNAs (14 upregulated and 102 downregulated) were identified and used for further study (Fig. 2g and Fig. 2h).

### Functional enrichment analysis

**Fig. 3:**
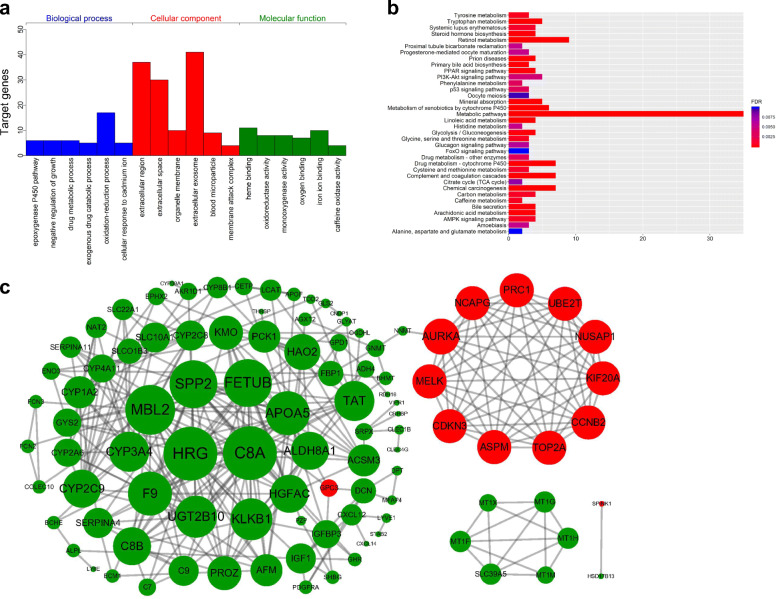
Functional annotation, pathway enrichment and PPI network analyses of the DEGs identified from the GEO and TCGA databases. **a** GO functional annotation; **b** KEGG pathway enrichment analysis; **c** PPI network, pink nodes represent upregulated genes, and green nodes represent downregulated genes; the node size is positively associated with the number of genes the node/gene can interact with

We performed GO functional annotations, KEGG pathway enrichment and PPI network analyses for 116 DEmRNAs using the DAVID, KOBAS and STRING databases, respectively. As a result of GO functional analysis, DEmRNAs were significantly enriched in 18 terms (*P*-value< 0.01), such as ‘extracellular exosome’, ‘extracellular region’, ‘extracellular space’ and ‘oxidation-reduction process’ (Fig. 3a). In terms of KEGG pathway analysis, the DEmRNAs were markedly enriched in ‘metabolic pathways’, ‘retinol metabolism’ and ‘chemical carcinogenesis’ terms (FDR < 0.01) (Fig. 3b). After the removal of the isolated and partially connected nodes, the PPI network containing a total of 101 nodes and 654 interactions was formulated according to a combined score > 0.4 (Fig. 3c).

### Construction of the ceRNA network

**Fig. 4:**
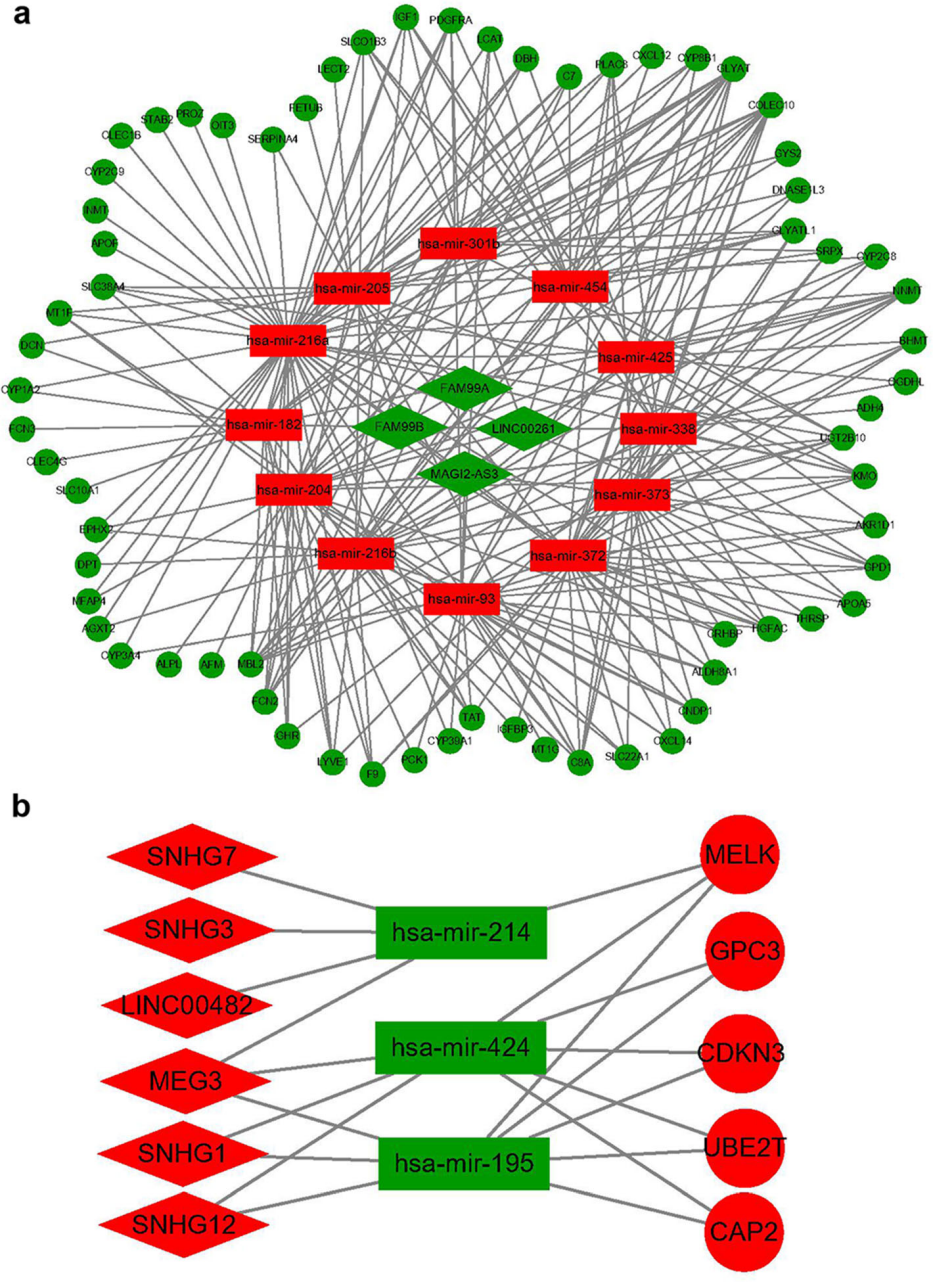
The lncRNA-miRNA-mRNA competing endogenous RNA networks for hepatocellular carcinoma. **a** The downregulated competing endogenous RNA network, including 4 downregulated lncRNAs, 12 upregulated miRNAs and 67 downregulated mRNAs; **b** The upregulated competing endogenous RNA network, including 6 upregulated lncRNAs, 3 downregulated miRNAs and 5 upregulated mRNAs. Diamonds represent lncRNAs; rectangles represent miRNAs; ellipses represent mRNAs; red indicates upregulated genes, and green represents downregulated genes

To obtain the target miRNAs of lncRNAs, the miRcode database was searched according to the 233 DElncRNAs, including 169 upregulated and 64 downregulated lncRNAs. To obtain the target mRNAs of miRNAs, miRanda and TargetScan were searched according to the 186 DEmiRNAs, including 163 upregulated and 23 downregulated miRNAs. First, downregulated DElncRNAs interacting with upregulated DEmiRNAs were retrieved from the miRcode database on the basis of their competitive relationship. Subsequently, downregulated DEmRNAs were searched in the miRanda and TargetScan databases according to upregulated DEmiRNAs because the mRNAs were negatively regulated by the miRNAs. As a result, 4 downregulated DElncRNAs capable of interacting with 12 upregulated DEmiRNAs and the corresponding 67 downregulated DEmRNAs were chosen to establish a visualized ceRNA network, named the ‘downregulated’ ceRNA network (Fig. 4a). Similarly, 6 upregulated DElncRNAs, 3 downregulated DEmiRNAs and 5 upregulated DEmRNAs were selected to construct another visualized ceRNA network, called the ‘upregulated’ ceRNA network (Fig. 4b). More details about lncRNA-miRNA and miRNA-mRNA interactions involved in the ceRNA networks are shown in the Table 1 and Table 2, respectively. To better understand the potential functions of the DEmRNAs in the regulatory ceRNA networks, we conducted GO functional annotation, KEGG pathway enrichment and PPI network analyses. The results are displayed in Fig. S4a, Fig. S4b and Fig. S4c.

**Table 1:** DEmiRNAs that is targeted by specific DElncRNAs in the ceRNA network

DElncRNA	DEmiRNA
**up-regulated**	**down-regulated**
LINC00482	miR-214
MEG3	miR-195,miR-214,miR-424
SNHG1	miR-195,miR-424
SNHG3	miR-214
SNHG7	miR-214
SNHG12	miR-195,miR-424
**down-regulated**	**up-regulated**
FAM99A	miR-205
FAM99B	miR-205
LINC00261	miR-182,miR-204,miR-216b,miR-301b,miR-338,miR-454
MAGI2-AS3	miR-93,miR-204,miR-216a,miR-216b,miR-372,miR-373,miR-425

Abbreviation: DElncRNAs, differentially expressed lncRNAs; DEmiRNAs, differentially expressed miRNAs

**Table 2:** DEmRNAs that is targeted by specific DEmiRNAs in the ceRNA network

DEmiRNA	DEmRNA
**down-regulated**	**up-regulated**
miR-195	CAP2,UBE2T,GPC3,MELK,CDKN3
miR-214	MELK
miR-424	CAP2,UBE2T,GPC3,MELK,CDKN3
**up-regulated**	**down-regulated**
miR-93	GHR,PDGFRA,LCAT,ALDH8A1,CYP39A1,GPD1,PCK1,COLEC10,MT1F,CXCL14, GLYAT,CYP3A4,TAT,C8A,KMO,SLC22A1,HGFAC,AKR1D1,NNMT,MT1G,CNDP1
miR-182	SLC10A1,F9,GHR,LYVE1,PDGFRA,SLCO1B3,FCN3,CYP8B1,CLEC4G,COLEC10, IGF1,CYP1A2,GLYAT,TAT,MBL2,SLC38A4,DCN,NNMT
miR-204	AFM,GHR,LYVE1,PDGFRA,UGT2B10,CRHBP,C7,PCK1,COLEC10,MT1F,CXCL14,GLYAT,FCN2,TAT,KMO,SLC22A1,CYP2C8,ALPL,DBH,DNASE1L3,MFAP4,EPHX2, NNMT,CNDP1
miR-205	LECT2,GLYATL1,C7,COLEC10,MT1F,GLYAT,FCN2,C8A,KMO,CYP2C8,DBH, SLC38A4,DCN,DNASE1L3,FETUB,SERPINA4,CNDP1
miR-216a	F9,GHR,AGXT2,LYVE1,PDGFRA,CYP2C9,LCAT,CYP39A1,GLYATL1,GPD1, PLAC8,C7,CYP8B1,COLEC10,OIT3,MT1F,IGF1,CYP1A2,GLYAT,FCN2,CYP3A4, KMO,MBL2,PROZ,HGFAC,STAB2,DBH,APOF,CLEC1B,SLC38A4,MFAP4,EPHX2,NNMT,SERPINA4,INMT,UGT2B10,ALDH8A1,TAT,C8A,DPT
miR-216b	F9,AGXT2,LYVE1,SLCO1B3,UGT2B10,ALDH8A1,BHMT,C7,CYP8B1,COLEC10, IGF1,GLYAT,FCN2,TAT,C8A,KMO,DPT,CYP2C8,MBL2,IGFBP3,EPHX2,NNMT, CNDP1
miR-301b	PDGFRA,GYS2,SLCO1B3,LCAT,UGT2B10,GLYATL1,PLAC8,C7,COLEC10,IGF1, CXCL12,GLYAT,FCN2,MBL2,SRPX
miR-338	APOA5,LYVE1,SLCO1B3,GPD1,PLAC8,COLEC10,IGF1,TAT,C8A,KMO,MBL2, OGDHL,HGFAC,DBH,ADH4,NNMT
miR-372	F9,APOA5,UGT2B10,ALDH8A1,BHMT,CRHBP,THRSP,GLYATL1,GPD1,PLAC8, COLEC10,CXCL14,GLYAT,C8A,SLC22A1,CYP2C8,MBL2,HGFAC,AKR1D1,SRPX,CNDP1
miR-373	F9,APOA5,UGT2B10,ALDH8A1,BHMT,CRHBP,THRSP,GLYATL1,GPD1,PLAC8, COLEC10,CXCL14,GLYAT,C8A,SLC22A1,CYP2C8,MBL2,HGFAC,AKR1D1,SRPX,CNDP1
miR-425	BHMT,CYP8B1,COLEC10,IGF1,GLYAT,C8A,KMO,MBL2,OGDHL,IGFBP3, AKR1D1,SLC38A4,DNASE1L3,NNMT
miR-454	PDGFRA,GYS2,SLCO1B3,LCAT,UGT2B10,GLYATL1,PLAC8,C7,COLEC10,IGF1, CXCL12,GLYAT,FCN2,MBL2,SRPX

Abbreviation: DEmiRNAs, differentially expressed miRNAs; DEmRNAs, differentially expressed mRNAs

### Screening of prognostic genes

**Fig. 5:**
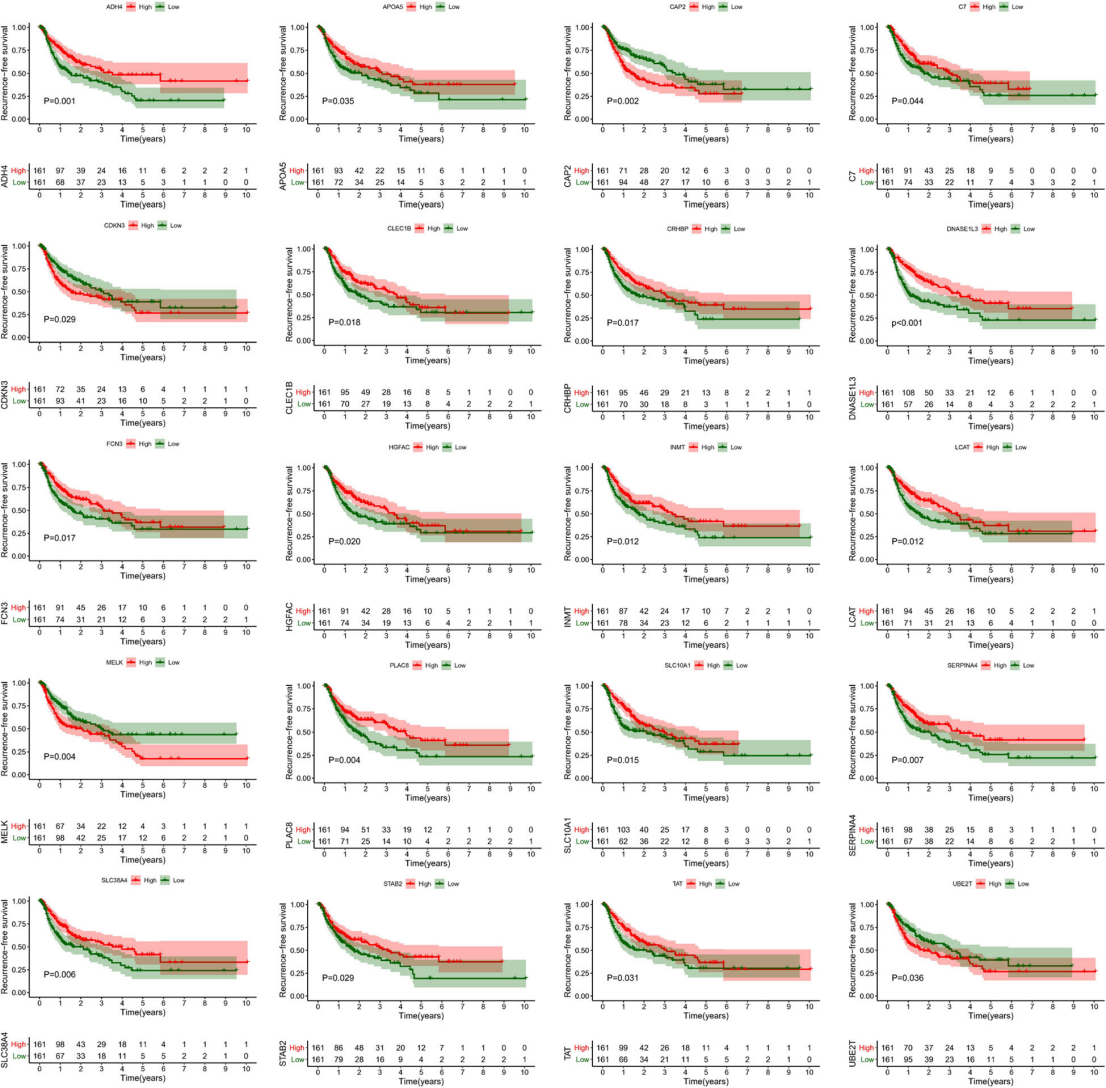
Kaplan-Meier curves of twenty genes associated with recurrence-free survival. The order of Kaplan-Meier curves of prognostic genes is as follows: ADH4, APOA5, CAP2, C7, CDKN3, CLEC1B, CRHBP, DNASE1L3, FCN3, HGFAC, INMT, LCAT, MELK, PLAC8, SLC10A1, SLE38A4, SERPINA4, STAB2, TAT, and UBE2T

**Fig. 6:**
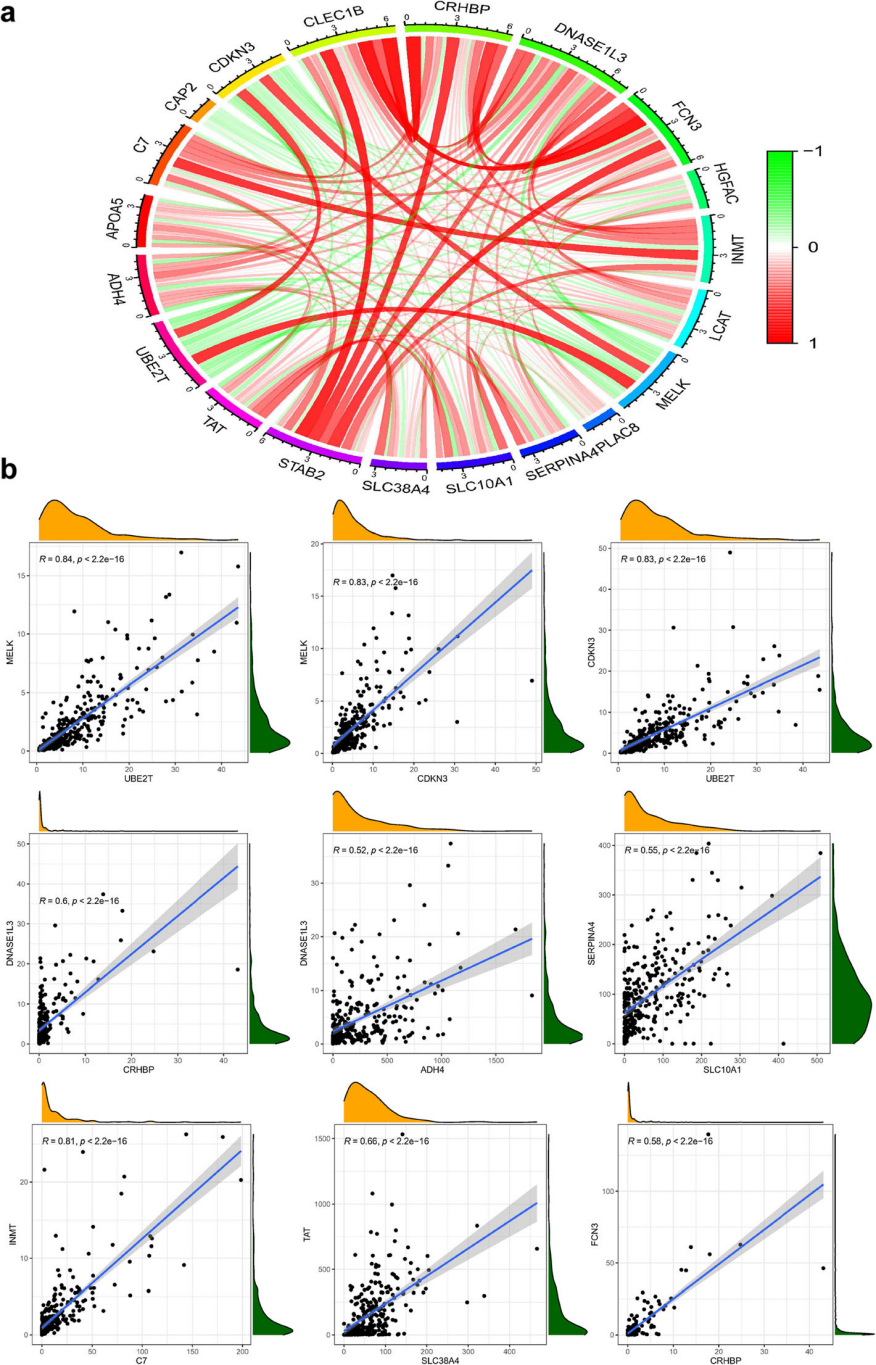
Co-expression of the 20 recurrence-related genes in HCC patients from the TCGA database. Pearson correlation analysis was performed with *p* < 0.05 being set as significant difference. **a** Total co-expression pattern between the 20 genes; **b** Representative co-expression genes with good correlation

According to the Kaplan-Meier analysis and log-rank test, a total of 20 DEmRNAs (ADH4, APOA5, CAP2, C7, CDKN3, CLEC1B, CRHBP, DNASE1L3, FCN3, HGFAC, INMT, LCAT, MELK, PLAC8, SLC10A1, SLE38A4, SERPINA4, STAB2, TAT, and UBE2T) involved in the ceRNA networks were identified as recurrence-related genes (*P*-value< 0.05, Fig. 5, See more details in Table S3). Based on the gene expression data retrieved from the TCGA database, 4 of the 20 DEmRNAs (CAP2, CDKN3, MELK and UBE2T) were upregulated in tumor tissues, and the remaining DEmRNAs were upregulated in adjacent normal tissues (Fig. S5). Interestingly, all four upregulated DEmRNAs were positively associated with recurrence, and all sixteen downregulated DEmRNAs were negatively associated with recurrence. In summary, the four upregulated DEmRNAs were found to be risk factors, and the sixteen downregulated DEmRNAs were protective factors. Combining the expression levels with survival analysis, however, they may all play harmful roles in tumorigenesis and progression. The expression correlation between genes identified as recurrence-related was shown in Fig. 6.

### Establishment of the prognostic signature

**Fig. 7:**
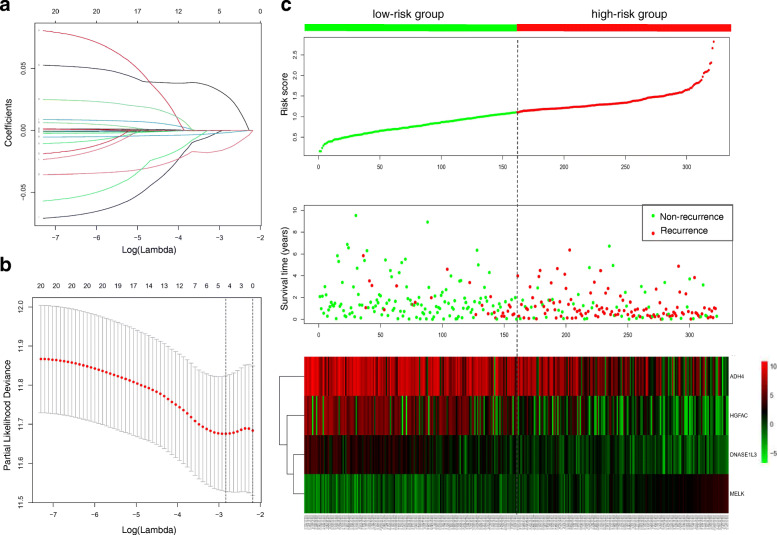
Construction of the prognostic signature for HCC based on the TCGA database. **a** LASSO regression coefficient profile of the 20 recurrence-related genes; **b** LASSO deviance profile of the 20 recurrence-related genes; **c** From top to bottom are the risk score distribution, recurrence status distribution, and heat map of genes in the prognostic signature between the low-risk and high-risk groups

Lasso-penalized Cox regression analysis using the “glmnet” R software package was conducted to select prognostic genes for formulate a multigene signature to predict RFS in patients with HCC. As a result, the best prognostic signature consisting of 4 DEmRNAs (ADH4, DNASE1L3, HGFAC and MELK) was established based on the minimum value of cross-validation error (Fig. 7a and Fig. 7b). The final RS formula was as follows: RS = (− 0.0037 × expression of ADH4) + (− 0.3131 × expression of DNASE1L3) + (− 0.0519 × expression of HGFAC) + (0.4626 × expression of MELK). The HCC patients were divided into the high-risk group and the low-risk group based on the median RS of the prognostic signature. The distributions of the RS, recurrence status and gene expression levels based on the prognostic signature between the low-risk and high-risk groups are shown in Fig. 7c. Patients with high RSs had shorter RFS times and were more likely to experience relapse. Note that the coefficients of three genes (ADH4, DNASE1L3 and HGFAC) were negative in this formula and one (MELK) positive, indicating that ADH4, DNASE1L3 and HGFAC might be protective factors and MELK risk factors, which is in accordance with previous survival analysis.

### Predictive performance in the TCGA cohort

**Fig. 8:**
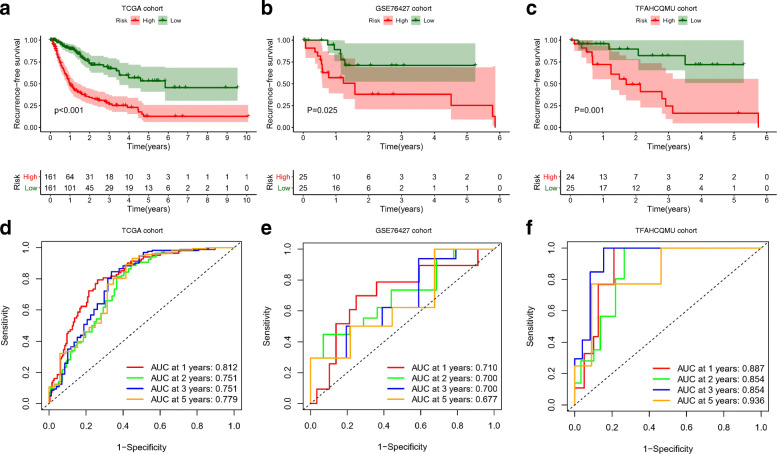
Performance and validation of the prognostic signature for HCC in different cohorts. Kaplan-Meier curves for the low-risk and high-risk groups: **a** The TCGA database; **b** The GSE76427 dataset; **c** The TFAHCQMU cohort; Time-dependent ROC curves for predicting HCC recurrence by the risk score: **d** The TCGA database; **e** The GSE76427 dataset; **f** The TFAHCQMU cohort

**Fig. 9:**
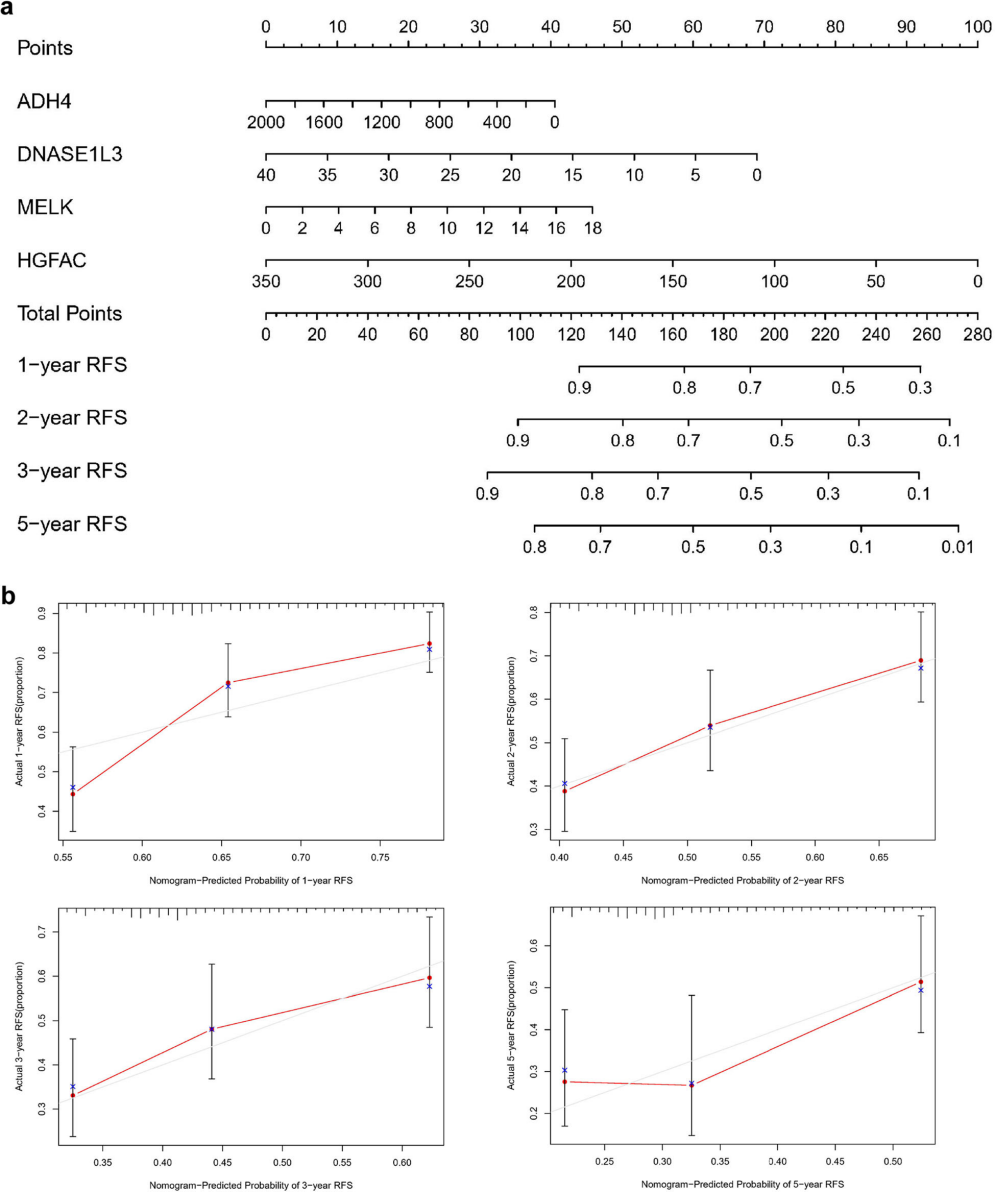
Nomogram of the prognostic signature for predicting HCC RFS at 1-, 2-, 3-, and 5-year in the TCGA database (*n* = 322)

There were 322 HCC patients with complete recurrence information, and 136 patients (42.24%) relapsed during the follow-up period. The basic clinical features of the HCC patients in the TCGA cohort are shown in Table S1. Kaplan-Meier analysis indicated that patients in the high-risk group had shorter RFS times than those in the low-risk group (*P*-value< 0.0001) and were more likely to experience relapse (Fig. 8a). The time-dependent AUCs of the prognostic signature for HCC in the TCGA cohort were 0.812, 0.751, 0.751 and 0.779 for 1-year, 2-year, 3-year, and 5-year RFS, respectively (Fig. 8d). The nomogram of the signature showed valuable and reliable probability for predict the RFS of HCC (Fig. 9). The potential relationships between the prognostic four-gene signature and other clinicopathological features was explored. The results indicated that the prognostic signature was significantly correlated with American Joint Committee on Cancer (AJCC) stage and vascular invasion (Table 3).

**Table 3:** Relationship between the prognostic four-gene signature and other clinicopathlogical features in the TCGA cohort and the TFAHCQMU cohort

Clinicopathological variables	TCGA cohort			TFAHCQMU cohort		
	Low risk	High risk	***P***-value	Low risk	High risk	***P***-value
Age			0.655			0.108
< 60	72	76		19	13	
≥60	89	85		6	11	
Gender			0.012*			0.675
Male	121	100		20	18	
Female	40	61		5	6	
Cirrhosis			0.862			0.308
Negative	64	51		13	9	
Positive	41	31		12	15	
AJCC stage			0.016*			0.049*
I-II	124	106		24	18	
III-IV	28	46		1	6	
Tumor grade			0.758			0.928
I-II	103	101		18	17	
III-IV	56	59		7	7	
Vascular invasion			0.028*			0.004**
Negative	105	81		22	12	
Positive	40	54		3	12	

TCGA, The Cancer Genome Atlas; TFAHCQMU, the First Affiliated Hospital of Chongqing medical university;

The median of the four-gene signature score was used as the cut-off values to divide HCC patients into the high-risk group and the low-risk group

Chi square test was used for comparison between two groups

*P-value< 0.05, **P-value< 0.01,***P-value< 0.001

### External validation of the signature

**Fig. 10:**
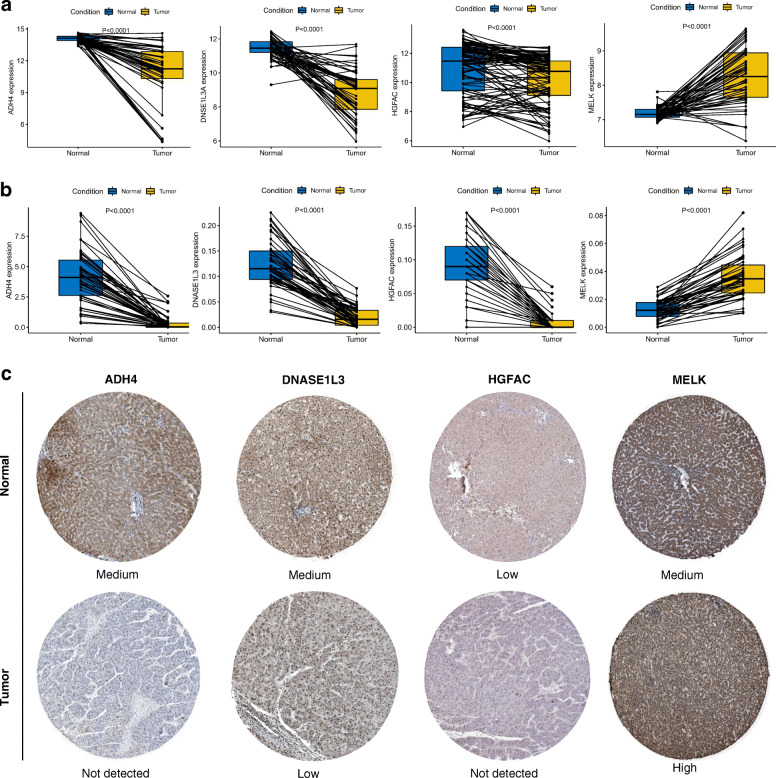
Gene expression of ADH4, DNASE1L3, HGFAC, and MELK. Expression at mRNA level between paired HCC tumor tissues and adjacent normal tissues: **a** In the GSE76427 dataset; **b** In the TFAHCQMU cohort; **c** Expression at protein level between HCC tumor tissues and normal tissues from the Human Protein Atlas (http://www.proteinatlas.org) online database

In the GSE76427 dataset, 20 HCC patients (38.46%) recurred during the follow-up period. In the TFAHCQMU cohort, 19 HCC patients (38.77%) experienced relapsed. The basic clinical features of the HCC patients in the TFAHCQMU cohort are shown in Table S1. Kaplan-Meier analysis indicated that patients in the low-risk group had longer RFS times and were less likely to relapse in both the GSE76427 dataset and the TFAHCQMU cohort (Fig. 8b and Fig. 8c). The time-dependent AUCs of the prognostic signature for HCC in the GSE76427 dataset and the TFAHCQMU cohort were 0.710, 0.700, 0.700 and 0.677 for 1-year, 2-year, 3-year, and 5-year RFS (Fig. 8e), 0.887, 0.854, 0.854 and 0.936 for 1-year, 2-year, 3-year, and 5-year RFS (Fig. 8f), respectively. The potential relationships between the prognostic four-gene signature and other clinicopathological features in the TFAHCQMU cohort indicated that the prognostic signature was significantly correlated with American Joint Committee on Cancer (AJCC) stage and vascular invasion (Table 3). The diagram of gene expression at mRNA level showed that there was a significant difference between HCC tumor tissues and paired adjacent normal tissues (Fig. 10a and Fig. 10b). Gene expression at protein level between HCC tumor tissues and normal tissues was representatively demonstrated in Fig. 10c.

### Independence assessment

Univariate and multivariate Cox regression analyses were successively carried out to assess the independence of prognostic factors for RFS in HCC patients. The results indicated that the prognostic signature and AJCC stage were independent risk factors for RFS in both the TCGA dataset and the TFAHCQMU cohort by multivariate Cox regression analysis (*P*-value< 0.05). Table 4 shows the results of univariate and multivariate Cox regression analyses of the four-gene signature and other prognostic factors for RFS in the TCGA cohort and TFAHCQMU cohort.

**Table 4:** Univariate and multivariate Cox regression analyses of the four-gene signature and other prognostic factors for recurrence-free survival in TCGA cohort and TFAHCQMU cohort

	Univariate analysis			Multivariate analysis		
Recurrence-free survival	HR	95%CI	***P***-value	HR	95%CI	***P***-value
TCGA cohort						
Age (≥60 vs < 60)	1.080	0.770–1.514	0.656	–	–	–
vGender (male vs female)	1.062	0.734–1.536	0.750	–	–	–
Cirrhosis (positive vs negative)	1.422	0.930–2.176	0.104	–	–	–
AJCC stage (III-IV vs I-II)	2.493	1.718–3.616	< 0.001***	2.776	1.736–4.440	< 0.001***
Tumor grade (III-IV vs I-II)	0.981	0.685–1.405	0.917	–	–	–
Vascular invasion (positive vs negative)	1.730	1.177–2.541	0.005**	1.113	0.741–1.672	0.606
Signature (high-risk vs low-risk)	3.107	1.783–5.951	< 0.001***	2.553	1.154–5.782	< 0.001***
TFAHCQMU cohort						
Age (≥60 vs < 60)	1.213	0.469–3.134	0.995	–	–	–
Gender (male vs female)	1.012	0.381–1.821	0.316	–	–	–
Cirrhosis (positive vs negative)	1.478	0.571–3.826	0.311	–	–	–
AJCC stage (III-IV vs I-II)	3.954	1.186–9.415	0.016*	2.598	1.546–7.109	0.042*
Tumor grade (III-IV vs I-II)	1.592	0.615–4.11	0.129	–	–	–
Vascular invasion (positive vs negative)	2.012	0.785–5.162	0.064	–	–	–
Signature (high-risk vs low-risk)	5.023	3.422–9.836	< 0.001***	3.289	2.821–8.481	0.002**

RFS,recurrence-free survival; TCGA, The Cancer Genome Atlas; TFAHCQMU, the First Affiliated Hospital of Chongqing medical university; CI,confidence interval; HR,hazard ratio

*P-value< 0.05, **P-value< 0.01, ***P-value< 0.001

### Establishment of a predictive nomogram

**Fig. 11:**
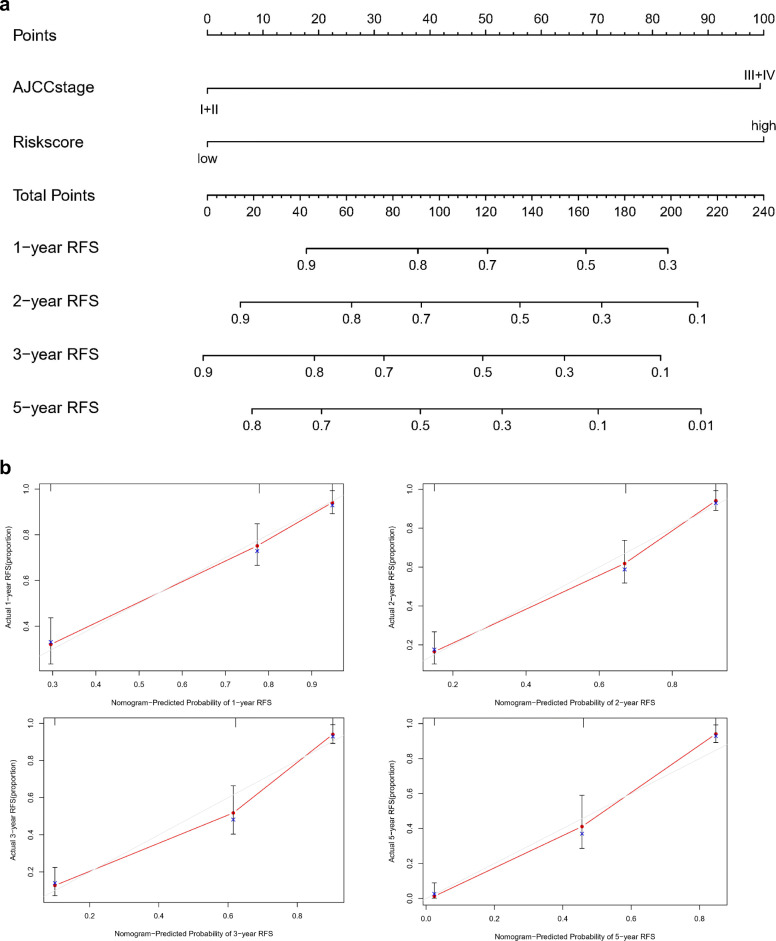
The integrated Nomogram consists of the four-gene signature and AJCC stage for predicting HCC RFS at 1-, 2-, 3-, and 5-year in the TCGA database

The nomogram, which included all independent factors identified by multivariate Cox regression analysis, was established. The results indicated that 1-, 2-, 3-, and 5-year RFS increased when risk scores declined, which is consistent with our previous findings, confirming the prognostic value of this risk nomogram. Combining the four-gene signature and clinicopathological feature (AJCC stage), the nomogram showed valuable and reliable predictive performance (Fig. 11).

## Discussion

HCC, which has high morbidity and mortality worldwide, is the main pathological type of liver cancer. Surgical treatments, the major interventional measures, can effectively improve the prognosis of early HCC patients; however, a large number of HCC patients are diagnosed at an advanced stage and are thus unsuitable for such treatments and eventually experience recurrence and metastasis. It remains a clinical challenge to identify patients who are at an early stage and predict patients who are at risk for recurrence after undergoing resection for HCC. Therefore, studies on the underlying molecular mechanisms of the tumorigenesis and progression of HCC are needed to identify reliable markers that can be used to assess the risk of recurrence and guide the development of personalized therapeutic strategies. As a result, HCC could be diagnosed at earlier stages, and such at-risk patients could receive close surveillance and novel interventional treatments. Moreover, for patients who are not at-risk but exceed the Milan criteria, liver transplantation may become the alternative choice of treatment.

Nevertheless, neither the widely accepted Barcelona Clinic Liver Cancer (BCLC) staging system nor the AJCC staging system for HCC includes molecular information, which can act as a complement to optimize therapeutic strategies and improve the clinical prognosis of HCC. Emerging evidence demonstrates that ceRNAs involved in signaling pathways are of significance in the tumorigenesis and progression of HCC, indicating that molecular markers based on the ceRNA network are equally important in the prediction of HCC recurrence.

Comprehensive analyses of large-scale microarray data and high-throughput sequencing data from public databases are often used to explore molecular biological mechanisms and identify potential molecular markers to help diagnose and predict prognosis. Gene signatures have allowed the accurate prediction of prognosis, and many studies have addressed prognostic prediction in HCC using array-based gene expression profiling. For example, Hoshida et al. [27] studied tissues from 307 HCC patients and discovered and validated a gene expression signature associated with OS with the use of a Cox regression model. As a result, they found that the gene expression profiles of the surrounding nontumor liver tissues were highly correlated with OS not only in a training set of 82 Japanese patients but also in an independent group of 225 patients from the United States and Europe. Villanueva et al. [28] assessed 287 HCC patients undergoing resection and tested genome-wide expression platforms using tumor (*n* = 287) and adjacent nontumor tissues to identify independent predictors of tumor recurrence based on Cox modeling. Finally, they developed a composite prognostic model for HCC recurrence that can predict early and overall recurrence in patients with HCC and complement findings from clinical and pathological analyses.

Certainly, many studies have explored some possible molecular regulatory pathways and feasible prognostic signatures of HCC to predict RFS, but few of them have constructed corresponding prognostic signatures based on ceRNA regulatory networks or validated the signature with other independent cohorts. Lv et al. [29], for instance, constructed a lncRNA-based classifier based on the expression profiles of seven lncRNAs (AL035661.1, PART1, AC011632.1, AC109588.1, AL365361.1, LINC00861, and LINC02084) to predict early recurrence in HCC after curative resection but did not establish a ceRNA network for HCC. Ye et al. [30] utilized Cox-penalized regression to develop a novel four-lncRNA (WARS2-IT1, AL359878.1, AL357060.1, and PART1) expression-based RS system for predicting the RFS of patients with HCC. Unfortunately, the RS systems were not further verified experimentally. Li et al. [31] partially compared the 1-year recurrence group (*n* = 56) with the nonrecurrence group (*n* = 60) of HCC patients from the TCGA database and constructed a hsa-mir-150-5p-centric ceRNA network and two effective prognostic nomogram models for predicting recurrence. Similarly, they failed to validate the results in an external cohort.

In the present study, three datasets with paired samples from studies on HCC were downloaded from the publicly available GEO database. The published original studies, from which the data were obtained, are as follows. Makowska et al. [20] found that gene expression profiling of HCC biopsies has limited potential to direct therapies that target specific driver pathways but can identify subgroups of patients with different prognoses. Grinchuk et al. [21] developed a prognostic stratification approach to identify common oncogenic pathways and significant prognostic variables in HCC patients with resectable primary tumors. Yang et al. [22] discovered and characterized an expanded landscape of lncRNAs based on high-throughput sequencing technology and bioinformatics analysis of matched samples from HCC patients.

To fully investigate the information of these datasets, multistep processing and integrated analyses were applied to reveal prognostic genes. Combined with the results of the TCGA database, a total of 116 dysregulated mRNAs (14 upregulated and 102 downregulated) were identified as DEGs and used for subsequent analyses (Fig. 3). In the GO functional analysis, the DEGs were predominantly enriched in extracellular areas and oxidation-reduction processes. With respect to KEGG pathway enrichment analysis, the DEGs were mainly enriched in metabolic-related pathways, which is in accordance with the findings of a previous study [32]. The visualized PPI network showed that the interactions between the DEGs were almost separately enriched in upregulated and downregulated genes. Subsequently, two biologically predicted ceRNA networks were constructed by comparing three RNA levels (lncRNAs, miRNAs and mRNAs) based on the competitive relationships of the ceRNA hypothesis to elucidate the interactions and regulatory mechanisms of the DEGs. The upregulated ceRNA network consisted of 6 upregulated DElncRNAs, 3 downregulated DEmiRNAs and 5 upregulated DEmRNAs, and the downregulated network included 4 downregulated DElncRNAs, 12 upregulated DEmiRNAs and 67 downregulated DEmRNAs. A total of 20 DEmRNAs involved in the ceRNA networks were found to be closely associated with recurrence by the Kaplan-Meier analysis and log-rank test using the gene expression profiles and survival information from the TCGA database, among which four upregulated DEmRNAs were risk factors and sixteen downregulated DEmRNAs were protective factors. Combining the expression levels with survival analysis, however, they all may play harmful roles in tumorigenesis and progression. Based on the 20 recurrence-related DEmRNAs, we adopted the LASSO-penalized regression method to successfully establish a four-gene signature (ADH4, DNASE1L3, HGFAC and MELK), which was assessed by time-dependent ROC curve analysis and presented a clear relationship with RFS. The AUCs of the prognostic signature for HCC in the TCGA cohort were 0.812, 0.751, 0.751 and 0.779 for 1-year, 2-year, 3-year, and 5-year RFS, respectively. The AUCs in the GSE76427 validation cohort were 0.710, 0.700, 0.700 and 0.677 for 1-year, 2-year, 3-year, and 5-year RFS, respectively. The AUCs in the TFAHCQMU validation cohort were 0.887, 0.854, 0.854 and 0.936 for 1-year, 2-year, 3-year, and 5-year RFS, respectively. Univariate and multivariate Cox analyses also proved that the RS system was a significant independent predictor for the RFS of patients with HCC. Therefore, the present study screened several recurrence-related mRNAs and developed a prognostic signature with datasets from the TCGA database that was further verified by using two independent external validation cohorts from GSE76427 and TFAHCQMU.

Certain recurrence-related genes identified in the present study have been reported to be cancer-related genes. More importantly, all the four genes included in the prognostic signature were also reported to be associated with the prognosis of HCC. Alcohol dehydrogenase 4 (ADH4) is an important member of the ADH family that metabolizes a wide variety of substrates, including ethanol and retinol. Wei RR et al. [33] found that the expression of ADH4 at both the mRNA and protein levels was markedly reduced in HCC tumor tissues. Similar to that in our study, HCC patients with lower ADH4 expression had shorter survival time, and multivariate Cox analysis showed that ADH4 expression was an independent predictor of prognosis. Liu XY et al. [34] comprehensively analyzed the prognostic implications related to ADH family genes in HCC using bioinformatic methods. As a result, they found that the expression of ADH4 was significantly downregulated in HCC tissues compared to normal tissues. Moreover, they identified ADH4 as an independent factor for the survival of HCC patients. In addition, high expression of ADH4, along with several other ADHs, was found to be significantly associated with an improved prognosis in HCC patients, and negatively regulates oncogenic signaling pathways. Luo J et al. [35] recently reported that the expressions of key alcohol-metabolizing enzymes are repressed in alcoholic hepatitis patients and revealed a new regulationary mechanism for ADH genes that the non-canonical positive regulation of miR-148a on ADH4. In short, miR-148a promotes ADH4 expression by directly binding to the coding sequence of ADH4 and increasing the mRNA stability via an AGO1-dependent manner proved by in vitro experiments using HepG2 cells, in turn, the secondary structure of ADH4 transcript affected the target accessibility and binding of miR-148a-3p, which provides new idea for the miRNA-mediated mechanisms underlying the expressions of alcohol-metabolizing enzymes. Deoxyribonuclease 1 like 3 (DNASE1L3) expression levels were significantly downregulated in numerous types of gastrointestinal cancer, and especially in HCC. Chen QY et al. [36] demonstrated that DNASE1L3 expression levels were frequently downregulated in tumor tissues compared with normal tissues, and were identified to be significantly associated with tumor size, thrombus formation, overall survival and disease-free survival of HCC patients. In addition, the ectopic expression of DNASE1L3 suppressed cell growth and inhibited the PI3K/AKT signaling pathway activation following C3a receptor agonist treatment. Zhang JJ et al. [37] established a comprehensive mRNA-miRNA-lncRNA triple ceRNA network, in which all RNAs, including DNASE1L3, were significantly linked to prognosis of patients with hepatocellular carcinoma. Wang S et al. [38] proved that DNASE1L3 is downregulated in both mRNA and protein levels in HCC tissues, compared with adjacent normal tissues. Patients with positive DNASE1L3 expression had significantly longer overall survival, compared with patients with negative expression. Moreover, Multivariate COX analysis revealed that positive DNASE1L3 expression, along with higher differentiation, is an independent prognostic factor. Hepatocyte growth factor activator (HGFAC), an activator of hepatocyte growth factor (HGF), has been previously reported to be involved in liver regeneration in response to injury and several types of cancers. Yin et al. [39] reported that HGFAC expression at the transcriptional and translational levels was decreased in liver cancer compared with normal tissues and patients with lower HGFAC expression level suffered shorter OS time. Fukushima T et al. [40] reviewed current knowledge regarding HGFAC-mediated proHGF activation and its roles in tissue regeneration and repair. Hepatocyte growth factor (HGF) is secreted as an inactive precursor (proHGF) and requires proteolytic activation to initiate HGF-induced signaling, while HGF activator (HGFAC) is a serum activator of proHGF and produces robust HGF activities in injured tissues. Xia et al. [41] previously performed gene expression profile analysis on HCC samples and identified maternal embryonic leucine zipper kinase (MELK) highly overexpressed, which was correlated with early recurrence and poor overall survival. They therefore further explored the functional roles of MELK and demonstrated that silencing MELK inhibited the cell growth, invasion, stemness and tumorigenicity of HCC cells by inducing apoptosis and mitosis, suggesting that MELK is a promising molecular target for therapeutic strategies against HCC [42]. Zhang X et al. [43] analyzed the therapeutic effect of targeted inhibition of MELK, named OTSSP167, on Glioblastoma multiforme (GBM). As a result, they found that OTSSP167 significantly inhibited cell proliferation, colony formation, invasion, and migration of GBM cells. Furthermore, OTSSP167 effectively prolonged the survival of tumor-bearing mice and inhibited tumor cell growth in in vivo mouse models. The treatment of OTSSP167 also reduced protein kinase B (AKT) phosphorylation levels, thereby disrupting the proliferation and invasion of GBM cells. In conclusion, MELK inhibition suppresses the growth of GBM by blocking AKT signals. Targeted inhibition of MELK may thus be potentially used as a novel treatment for not only GBM, but also other diseases. However, the remaining recurrence-related genes identified in the current study need further investigation and exploration.

In contrast to previous studies, the present study has several strengths. First, we used large-scale microarray and sequencing data of HCC patients from both the GEO and TCGA databases. Second, we included paired samples from three GEO datasets to eliminate errors among different patients since each gene expression level varied substantially in different patients. Third, compared with previous studies that constructed only one mixed ceRNA network, we separately constructed two predictable ceRNA networks by comparing three RNA levels (lncRNAs, miRNAs and mRNAs) based on the competitive relationships of the ceRNA hypothesis. Fourth, we not only established a prognostic signature but also conducted independent external validations, which guaranteed that the results were reasonable and reliable. The results presented in this paper were based on sufficient samples, rigorous processes and appropriate methodology, but this study inevitably has several limitations. First, a larger group of samples and longer follow-up period should be used. Second, although the AUCs for the RS of recurrence were more than 0.7, they were still relatively low. Finally, further in-depth study on the molecular mechanisms of the identified ceRNA networks needs to be performed to verify our work.

## Conclusions

In conclusion, the present study identified a number of cancer-specific and recurrence-related genes by performing an integrated analysis of large-scale gene expression profiles from the GEO and TCGA databases. The two predictive lncRNA-miRNA-mRNA ceRNA networks may provide guidance for further studies on the tumorigenesis and progression mechanisms underlying HCC. The prognostic signature established by this paper shows promising prospects in clinical application and can probably help with early diagnosis and guide personalized treatments.

## Supplementary Information

**Additional file 1 Fig. S1** Heatmaps of the top 200, according to adjusted *P*-value, DEmRNAs identified from the TCGA database.

**Additional file 2 Fig. S2** Heatmaps of 233 DElncRNAs identified from the TCGA database.

**Additional file 3 Fig. S3** Heatmaps of 186 DEmiRNAs identified from the TCGA database.

**Additional file 4 Fig. S4** Functional analyses of the DEGs involved in the ceRNA networks. **a** GO functional annotation; **b** KEGG pathway enrichment analysis; **c** PPI network.

**Additional file 5 Fig. S5** Expression levels of the 20 genes associated with RFS between HCC tumor tissues and adjacent normal tissues in the TCGA database. The order of genes is as follows: ADH4, APOA5, CAP2, C7, CDKN3, CLEC1B, CRHBP, DNASE1L3, FCN3, HGFAC, INMT, LCAT, MELK, PLAC8, SLC10A1, SLE38A4, SERPINA4, STAB2, TAT, and UBE2T.

**Additional file 6.**

**Additional file 7.**

**Additional file 8.**

## Data Availability

The datasets generated and analyzed during the current study are available in the Gene Expression Omnibus (https://www.ncbi.nlm.nih.gov/geo/) and The Cancer Genome Atlas (TCGA) databases (https://portal.gdc.cancer.gov/) and are available from the corresponding author on reasonable request.

## Abbreviations

HCC: hepatocellular carcinoma
RFS: recurrence-free survival
OS: overall survival
ceRNA: competing endogenous RNA
MREs: miRNA response elements
DEGs: differentially expressed genes
DElncRNAs: differentially expressed long noncoding RNAs
DEmiRNAs: differentially expressed microRNAs
DEmRNAs: differentially expressed messenger RNAs
ROC: receiver operating characteristic
AUC: area under the ROC curve
TFAHCQMU: The First Affiliated Hospital of Chongqing Medical University
ADH4: Alcohol dehydrogenase 4
DNASE1L3: deoxyribonuclease 1 like 3
HGFAC: Hepatocyte growth factor activator
MELK: maternal embryonic leucine zipper kinase
GEO: Gene Expression Omnibus
TCGA: The Cancer Genome Atlas
DAVID: Database for Annotation, Visualization, and Integrated Discovery
KOBAS: KEGG Orthology Based Annotation System
KEGG: Kyoto Encyclopedia of Genes and Genomes
STRING: The Search Tool for the Retrieval of Interacting Genes
AJCC: American Joint Committee on Cancer
BCLC: Barcelona Clinic Liver Cancer
