# Supplementary figures and images for: Comprehensive analyses of competing endogenous RNA networks reveal potential biomarkers for predicting hepatocellular carcinoma recurrence
Source: BMC Cancer. 2021 Apr 20;21:436. doi: 10.1186/s12885-021-08173-0 (PMC8058997; doi:10.1186/s12885-021-08173-0)

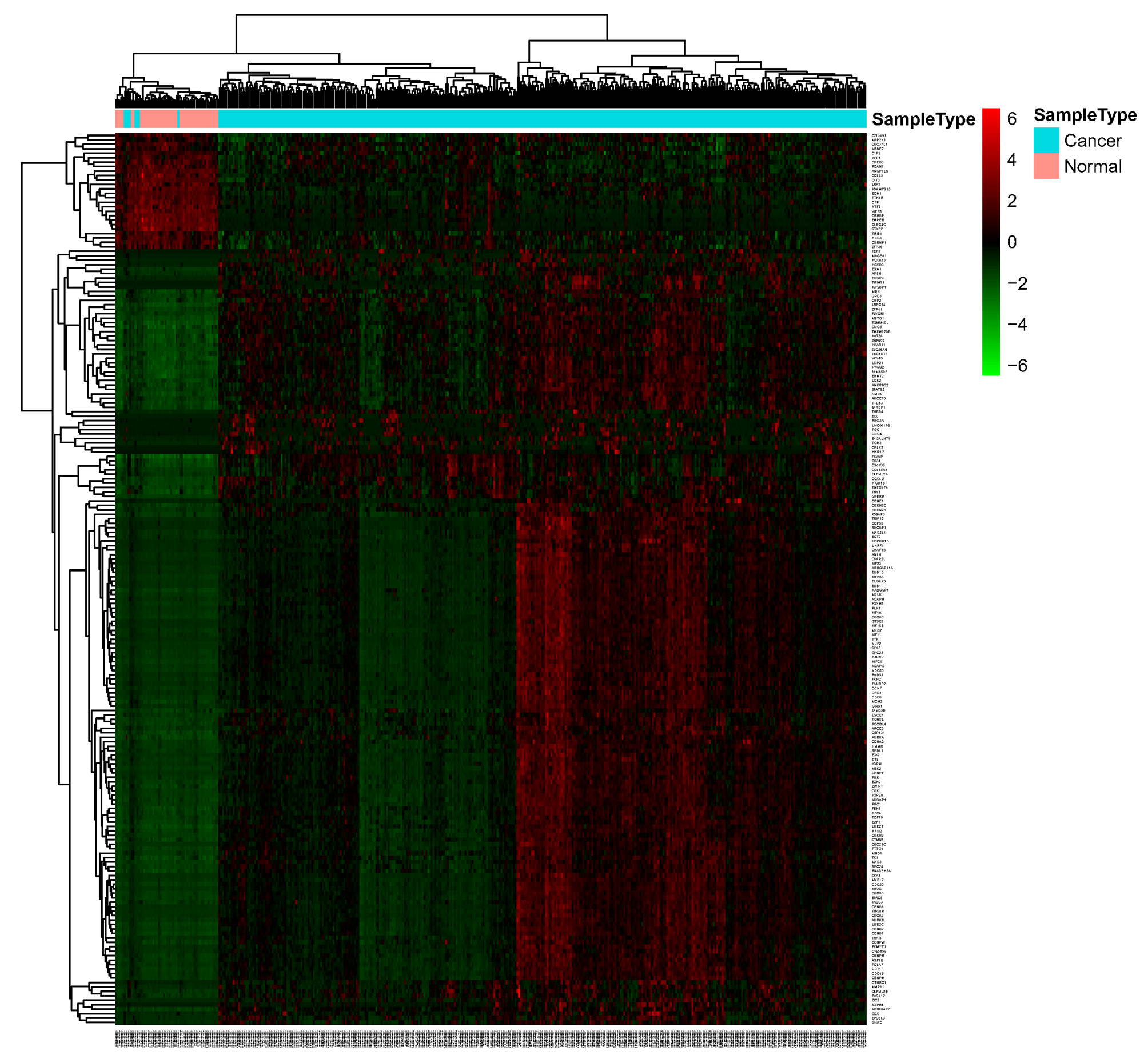

Supplement: Supplementary file 1 — Additional file 1 Fig. S1 Heatmaps of the top 200, according to adjusted P-value, DEmRNAs identified from the TCGA database. [file 12885_2021_8173_MOESM1_ESM.tiff]

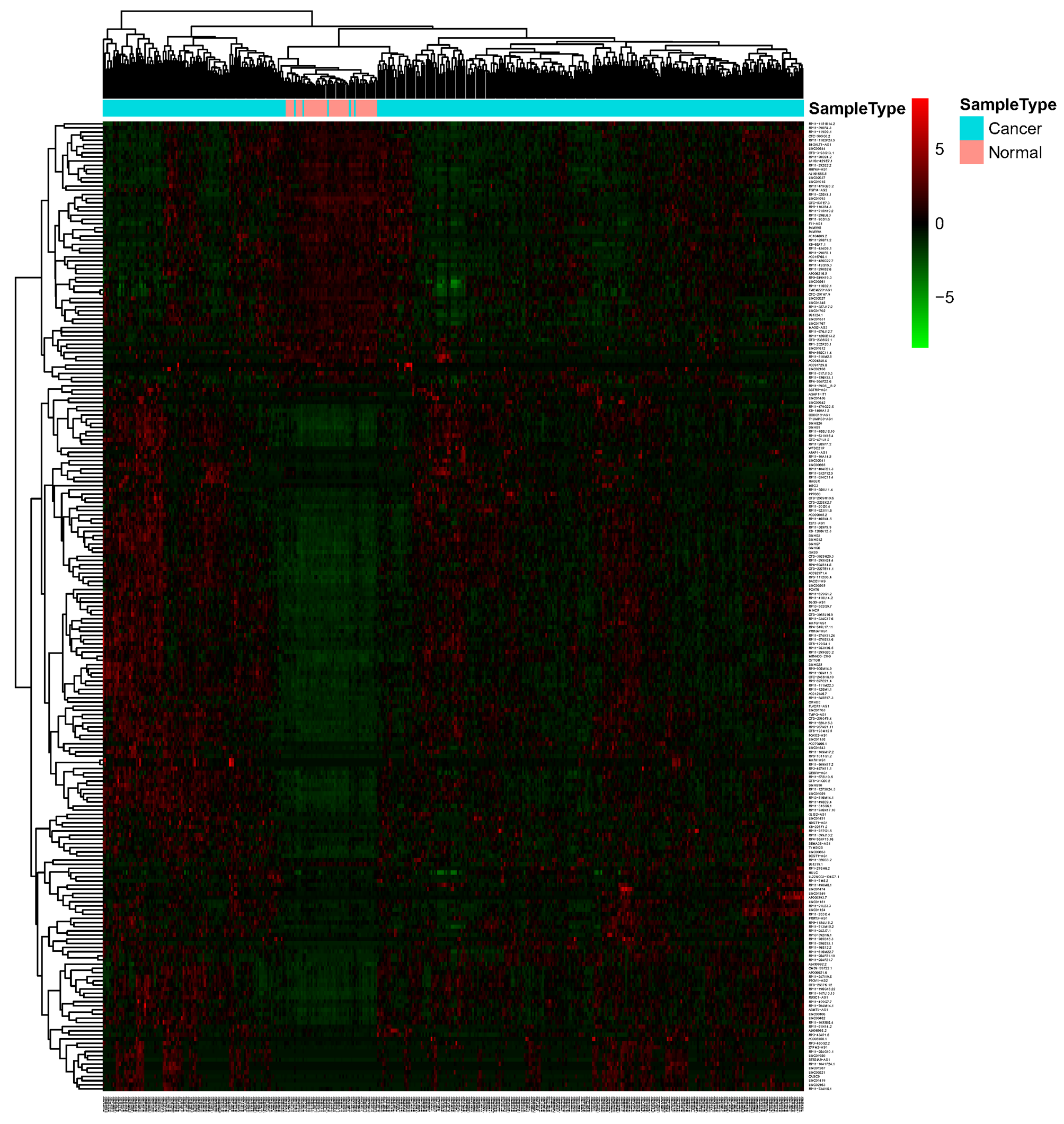

Supplement: Supplementary file 2 — Additional file 2 Fig. S2 Heatmaps of 233 DElncRNAs identified from the TCGA database. [file 12885_2021_8173_MOESM2_ESM.tiff]

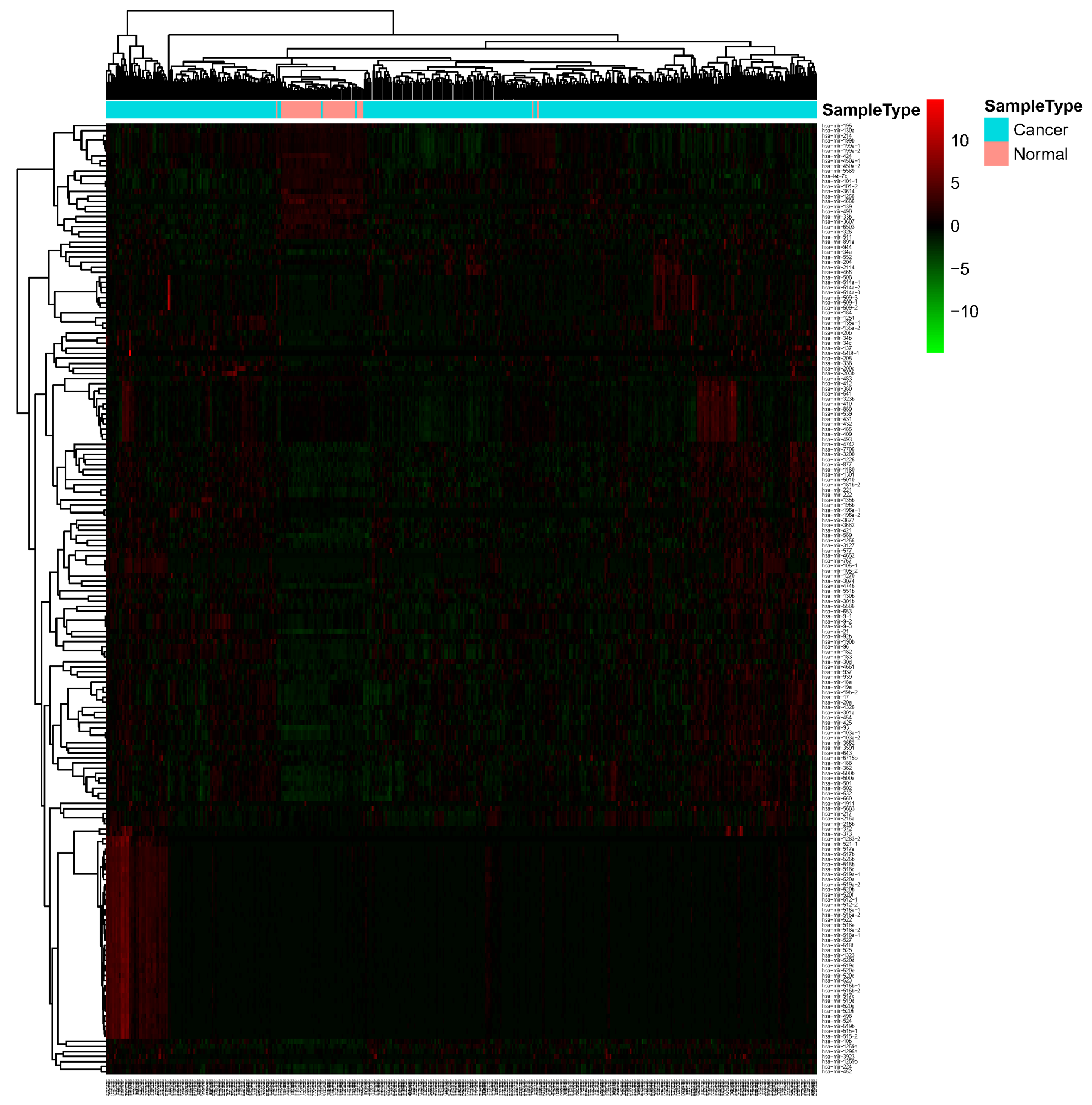

Supplement: Supplementary file 3 — Additional file 3 Fig. S3 Heatmaps of 186 DEmiRNAs identified from the TCGA database. [file 12885_2021_8173_MOESM3_ESM.tiff]

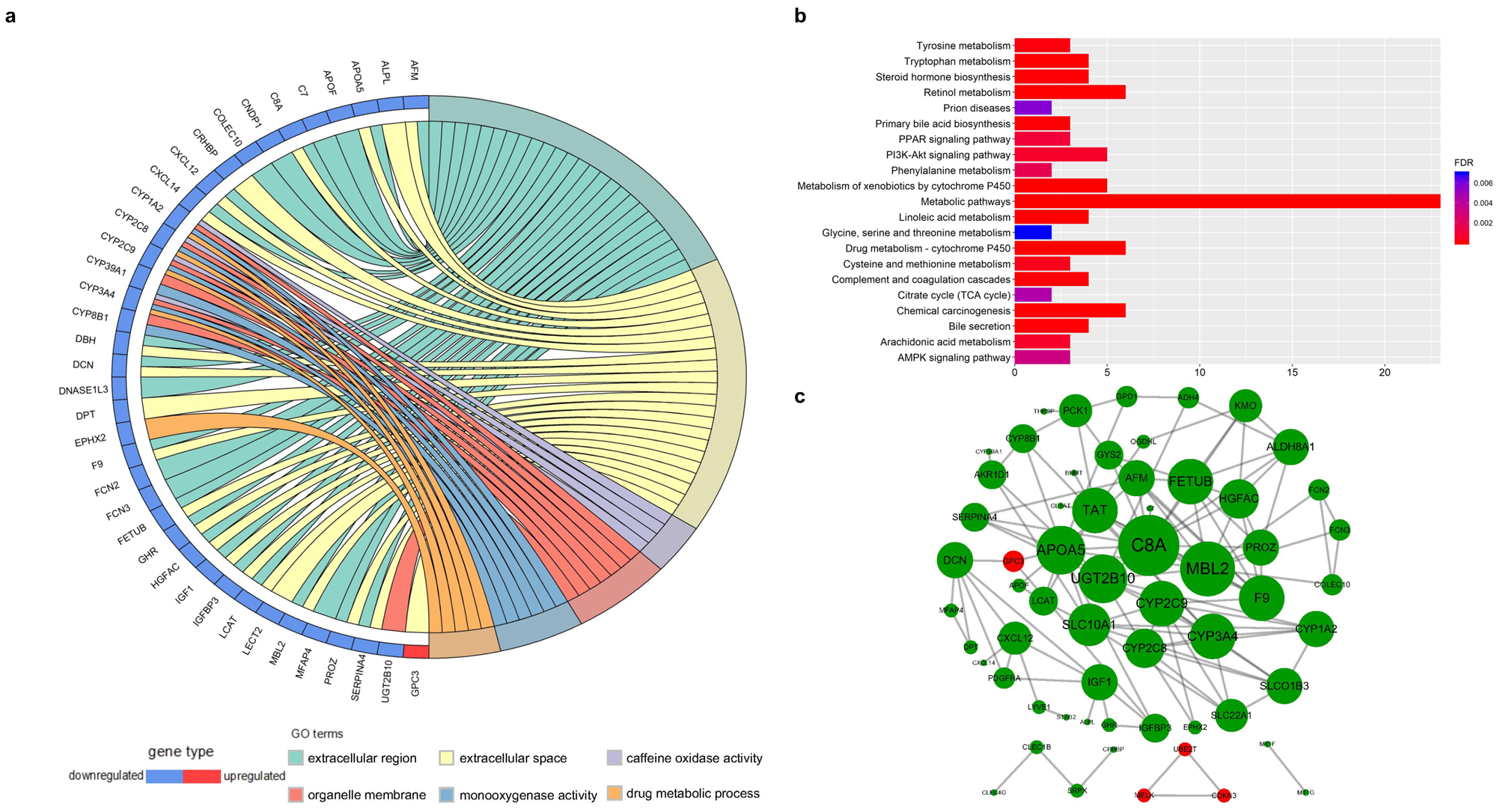

Supplement: Supplementary file 4 — Additional file 4 Fig. S4 Functional analyses of the DEGs involved in the ceRNA networks. a GO functional annotation; b KEGG pathway enrichment analysis; c PPI network. [file 12885_2021_8173_MOESM4_ESM.tiff]

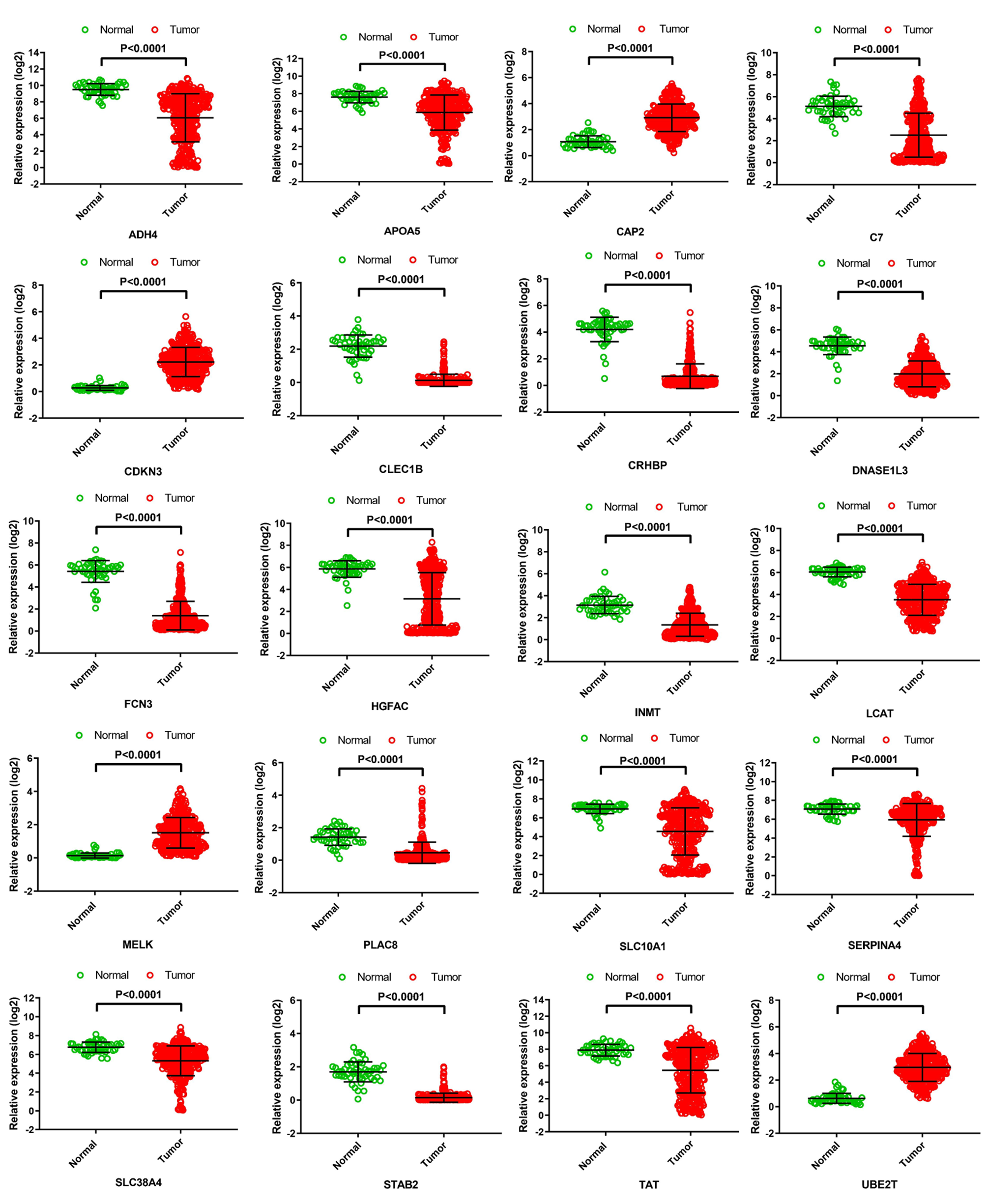

Supplement: Supplementary file 5 — Additional file 5 Fig. S5 Expression levels of the 20 genes associated with RFS between HCC tumor tissues and adjacent normal tissues in the TCGA database. The order of genes is as follows: ADH4, APOA5, CAP2, C7, CDKN3, CLEC1B, CRHBP, DNASE1L3, FCN3, HGFAC, INMT, LCAT, MELK, PLAC8, SLC10A1, SLE38A4, SERPINA4, STAB2, TAT, and UBE2T. [file 12885_2021_8173_MOESM5_ESM.tiff]
