# Supplementary material for: Comprehensive analyses of competing endogenous RNA networks reveal potential biomarkers for predicting hepatocellular carcinoma recurrence
Source: BMC Cancer. 2021 Apr 20;21:436. doi: 10.1186/s12885-021-08173-0 (PMC8058997; doi:10.1186/s12885-021-08173-0)
Supplement: Supplementary file 6 — Additional file 6. [file 12885_2021_8173_MOESM6_ESM.docx]

Table S1. Clinicopathological characteristics for the HCC patients in the TCGA cohort and TFAHCQMU cohort.

| **Clinicopathological variables** | **TCGA cohort**  **(n=322)** | **TFAHCQMU cohort**  **(n=49)** |
| --- | --- | --- |
| Age |  |  |
| <60 | 145 | 32 |
| ≥60 | 177 | 17 |
| Gender |  |  |
| Male | 221 | 38 |
| Female | 101 | 11 |
| Cirrhosis |  |  |
| Negative | 115 | 22 |
| Positive | 72 | 27 |
| AJCC stage |  |  |
| Ⅰ-Ⅱ | 230 | 42 |
| Ⅲ-Ⅳ | 74 | 7 |
| Tumor grade |  |  |
| Ⅰ-Ⅱ | 204 | 35 |
| Ⅲ-Ⅳ | 115 | 14 |
| Vascular invasion |  |  |
| Negative | 186 | 34 |
| Positive | 94 | 15 |

Abbreviations: TFAHCQMU, the First Affiliated Hospital of Chongqing Medical University; AJCC,American Joint Committee on Cancer.
