# Supplementary material for: Comprehensive analyses of competing endogenous RNA networks reveal potential biomarkers for predicting hepatocellular carcinoma recurrence
Source: BMC Cancer. 2021 Apr 20;21:436. doi: 10.1186/s12885-021-08173-0 (PMC8058997; doi:10.1186/s12885-021-08173-0)
Supplement: Supplementary file 7 — Additional file 7. [file 12885_2021_8173_MOESM7_ESM.docx]

Table S2. Primers used for quantitative real-time PCR.

| **Gene** | **Forward sequences** | **Reverse sequences** |
| --- | --- | --- |
| GAPDH | 5′-GAAGGTGAAGGTCGGAGTCAACG-3′ | 5′-TGCCATGGGTGGAATCATATTGG-3′ |
| ADH4 | 5′-AGTTCGCATTCAGATCATTGCT-3′ | 5′-CTGGCCCAATACTTTCCACAA-3′ |
| DNASE1L3 | 5′-AGCCCTTTGTGGTCTGGTTC-3′ | 5′-TCCTTAACGGATGTCTCTGGG-3′ |
| HGFAC | 5′-GTGTGCCACAACTCACAACTA-3′ | 5′-GGTCCTGGGTATTGGAGCA-3′ |
| MELK | 5′-TCTCCCAGTAGCATTCTGCTT-3′ | 5′-TGATCCAGGGATGGTTCAATAGA-3′ |
