# Supplementary material for: Comprehensive analyses of competing endogenous RNA networks reveal potential biomarkers for predicting hepatocellular carcinoma recurrence
Source: BMC Cancer. 2021 Apr 20;21:436. doi: 10.1186/s12885-021-08173-0 (PMC8058997; doi:10.1186/s12885-021-08173-0)
Supplement: Supplementary file 8 — Additional file 8. [file 12885_2021_8173_MOESM8_ESM.docx]

Table S3. Information of twenty genes associated with recurrence-free survival identified by Kaplan-Meier curve analysis and log-rank test in TCGA cohort.

| **Gene** | **Expression** | **1-year RFS** | **2-year RFS** | **3-year RFS** | **5-year RFS** | **Medium RFS**  **(year)** | **P-value** |
| --- | --- | --- | --- | --- | --- | --- | --- |
| ADH4 | High | 77.58% | 61.05% | 53.90% | 47.85% | 3.523 | 0.001** |
|  | Low | 57.75% | 47.30% | 40.69% | 20.35% | 1.471 |  |
| APOA5 | High | 76.87% | 59.40% | 51.39% | 37.44% | 3.096 | 0.035* |
|  | Low | 61.39% | 51.47% | 42.93% | 27.95% | 2.126 |  |
| CAP2 | High | 58.30% | 42.35% | 36.30% | 26.95% | 1.384 | 0.002** |
|  | Low | 76.45% | 66.13% | 56.10% | 36.85% | 3.523 |  |
| C7 | High | 75.90% | 59.95% | 51.70% | 39.05% | 3.096 | 0.044* |
|  | Low | 62.70% | 48.95% | 43.45% | 25.30% | 1.751 |  |
| CDKN3 | High | 61.05% | 47.83% | 42.35% | 25.81% | 1.537 | 0.029* |
|  | Low | 76.45% | 61.05% | 50.05% | 38.50% | 3.096 |  |
| CLEC1B | High | 75.92% | 62.15% | 54.76% | 35.91% | 3.504 | 0.018* |
|  | Low | 62.03% | 46.15% | 38.93% | 30.54% | 1.622 |  |
| CRHBP | High | 77.91% | 58.89% | 51.27% | 37.95% | 3.060 | 0.017* |
|  | Low | 60.44% | 47.85% | 42.94% | 23.10% | 1.605 |  |
| DNASE1L3 | High | 79.75% | 65.45% | 55.64% | 40.15% | 3.523 | <0.001*** |
|  | Low | 52.80% | 42.37% | 36.85% | 22.04% | 1.096 |  |
| FCN3 | High | 75.93% | 62.71% | 53.90% | 36.85% | 3.096 | 0.017* |
|  | Low | 60.55% | 46.75% | 40.15% | 29.42% | 1.605 |  |
| HGFAC | High | 76.49% | 63.25% | 54.92% | 37.06% | 3.367 | 0.020* |
|  | Low | 60.48% | 46.83% | 39.12% | 28.05% | 1.551 |  |
| INMT | High | 75.35% | 62.15% | 54.08% | 41.25% | 3.367 | 0.012* |
|  | Low | 63.25% | 48.77% | 39.05% | 22.58% | 1.742 |  |
| LCAT | High | 76.45% | 64.50% | 52.49% | 36.30% | 3.367 | 0.012* |
|  | Low | 58.21% | 43.45% | 41.09% | 27.56% | 1.537 |  |
| MELK | High | 59.96% | 50.25% | 43.54% | 17.05% | 2.066 | 0.004** |
|  | Low | 78.10% | 59.38% | 49.53% | 42.91% | 2.967 |  |
| PLAC8 | High | 73.72% | 63.11% | 58.85% | 40.15% | 3.923 | 0.004** |
|  | Low | 61.05% | 43.41% | 33.04% | 23.17% | 1.622 |  |
| SLC10A1 | High | 80.30% | 57.32% | 51.15% | 36.84% | 2.967 | 0.015* |
|  | Low | 55.49% | 51.13% | 45.12% | 27.50% | 2.126 |  |
| SERPINA4 | High | 75.90% | 59.95% | 53.81% | 41.27% | 3.504 | 0.007** |
|  | Low | 58.87% | 50.60% | 38.94% | 25.36% | 2.066 |  |
| SLC38A4 | High | 75.96% | 57.24% | 53.35% | 41.88% | 3.981 | 0.006** |
|  | Low | 59.84% | 50.10% | 40.31% | 24.20% | 2.126 |  |
| STAB2 | High | 72.64% | 61.69% | 53.42% | 42.79% | 3.367 | 0.029* |
|  | Low | 64.35% | 47.28% | 40.15% | 18.70% | 1.742 |  |
| TAT | High | 75.95% | 60.50% | 50.65% | 36.54% | 3.096 | 0.031* |
|  | Low | 58.85% | 50.19% | 43.94% | 30.33% | 2.126 |  |
| UBE2T | High | 60.07% | 49.59% | 42.35% | 26.75% | 1.742 | 0.036* |
|  | Low | 73.90% | 57.49% | 53.95% | 39.05% | 3.096 |  |

RFS,recurrence-free survival; TCGA, The Cancer Genome Atlas.

*P-value<0.05, **P-value<0.01, ***P-value<0.001.
